# Recent advances in the positive role of *Clostridium* in autoimmune diseases: as a crucial regulator of restoring immune-metabolic equilibrium

**DOI:** 10.3389/fimmu.2026.1730351

**Published:** 2026-03-25

**Authors:** Jing-Yi Zhan, Wen-Ru Wang, Tian Zhan, Jia-Yi Yang, Ying Liang, Ren-Huan Yu, Xin-Hui Wang

**Affiliations:** 1 Department of Nephrology, Xiyuan Hospital of China Academy of Chinese Medical Sciences, Beijing, China; 2 Graduate School of China Academy of Chinese Medical Sciences, Beijing, China

**Keywords:** autoimmune diseases, butyrate, clostridium, immune interaction, immunotherapy

## Abstract

Autoimmune diseases (ADs) are a group of conditions characterized by an overactive immune response that damages one or more organs or tissues of the body. Emerging evidence highlights a critical association between reduced abundance of beneficial *Clostridium* and the pathogenesis of ADs. Studies show that certain *Clostridium*, such as *Clostridium butyricum* (*CB*) and *Faecalibacterium prausnitzii* (*FP*), are almost depleted in multiple ADs. This depletion is associated with impaired immune homeostasis, disrupted intestinal barrier integrity, and heightened inflammation. In diseases such as inflammatory bowel disease (IBD), rheumatoid arthritis (RA), and type 1 diabetes (T1D), restoration of *Clostridium* species or their metabolites may alleviate disease severity. Mechanistically, *Clostridium* probiotics may modulate ADs via multiple therapeutic mechanisms, including regulating gut microbiota composition, replenishing beneficial metabolites to restore intestinal barrier integrity, correcting dysregulated immune cell differentiation and homeostasis, and balancing pro- and anti-inflammatory cytokine levels. Our review synthesizes current evidence to delineate the immunomodulatory mechanisms by which *Clostridium* and its metabolites influence ADs. These findings underscore the therapeutic potential of *Clostridium*-based interventions in reestablishing immune-metabolic equilibrium in ADs.

## 1 Introduction

Autoimmune diseases (ADs) arise from the immune system’s aberrant response against self-tissues, encompassing diverse conditions such as rheumatoid arthritis (RA), systemic lupus erythematosus (SLE), inflammatory bowel disease (IBD), type 1 diabetes (T1D), and primary biliary cholangitis (PBC) (1, 2). With a global prevalence of 3%–5% and rising incidence rates (particularly in industrialized nations), ADs collectively represent a major public health burden (1, 3). Their clinical management remains challenging due to heterogeneous pathogenesis, limited targeted therapies, and high treatment resistance (1, 4, 5). Although scientists have not yet fully elucidated the pathogenesis of autoimmune diseases, external and internal predisposing factors have been extensively analyzed and debated (6–9).

Mounting evidence suggests that gut microbiota and its metabolites play a key role in host health and diverse diseases, such as obesity, depression, sclerosis, kidney disease, and dialysis (10–15). As the body’s largest digestive organ, the gut is continuously exposed to the external environment (16, 17). Due to its unique architecture, the intestinal tract is inherently susceptible to stimulation by exogenous agents, including antigens, viruses, and bacteria (18, 19). To defend against pathogenic stimuli and maintain homeostasis, the gut has evolved specialized physiological structures with region-specific immune functions (20, 21). The immunological function of the intestine is primarily constituted by and critically regulated by various microbial populations and their metabolites (22, 23). Diverse bacteriocins, pathogen-associated molecular patterns (PAMPs), and metabolites, secreted by intestinal flora, have activated the capacity of the intestinal immune system mainly through the medium of adhering to the epithelium, binding pattern recognition receptors (PRRs), to achieve the aim of influencing the onset and progression of the disease (24–26). Notably, gut dysbiosis, including reduced beneficial taxa, pathogen expansion, or loss of diversity, will trigger intestinal barrier disruption (27–29). In parallel, intestinal barrier dysfunction contributes to the insidious development of systemic inflammation and immune dysregulation (30). This process, combined with other pathogenic factors, collectively underlies the high prevalence and clinical management difficulties of autoimmune disorders (31). Reversing the damage induced by microbial imbalance has become a recent research focus.

At present, in the interest of reshaping the disordered intestinal immune activity in the disease state and alleviating the disease, people propose a series of flora-related immunotherapies based on the close interaction between the intestinal immune system and the flora (32–34).

*Clostridium*, a genus of anaerobic, Gram-positive, thick-walled bacteria, constitutes a dominant component of the healthy gut microbiota (35). Its presence is essential for intestinal environmental stability (36), and specific beneficial *Clostridium* species may confer clinical benefits across multiple ADs (37). This review delineates the therapeutic potential of intestinal clostridia (as probiotics) in ADs and details their mechanisms of action, including (i) gut barrier reinforcement, (ii) microbial community modulation, (iii) correcting immune dysregulation, and (iv) modulating inflammatory cytokine levels.

## 2 Classification and sources of *Clostridium* as probiotics

The intestinal clostridia as probiotics mainly contain *Clostridium butyricum* (*CB*), *Faecalibacterium prausnitzii* (*FP*), *Clostridium cocleatum*, etc. (38). Most of them originate from feces, and a small proportion from the gut (39). The beneficial intestinal clostridia species primarily involved in this text and their sources are listed in **Table 1**. The literature retrieval strategy is provided in **Supplementary File 1**.

**Table 1:** Common beneficial *Clostridium* species and their main sources.

Designation	Source	Designation	Source
*Clostridium butyricum*	Feces	*Clostridium cluster XI*	Intestinal tract
*Faecalibacterium prausnitzii*	Feces	*Clostridium cluster XlVa*	Intestinal tract
*Clostridium nexile*	Feces	*Clostridium cluster XlVb*	Intestinal tract
*Clostridium saccharogumia*	Feces	*Clostridium cluster XVIII*	Intestinal tract
*Clostridium leptum*	Feces	*Clostridium clusters IV*	Intestinal tract
*Clostridium lavalense*	Feces	*Clostridium cocleatum*	Intestinal tract

*CB* is a typical butyrate-producing anaerobic bacterium that colonizes the human gut naturally (40). As the “symbiont,” *CB* has features such as consuming incompletely digested dietary fibers and manufacturing short-chain fatty acids (SCFAs) (41, 42), primarily comprising acetate, propionate, and butyrate (43–45). Butyrate can effectively promote the growth of normal gut flora and inhibit the proliferation of pathogenic microbes (46–50). Butyrate can also provide energy for gut epithelial cells and improve the integrity of gut mucosa by a accelerating their growth and polarization, thus avoiding risky conditions or *in vitro* pathogenic factors that can cause inflammatory lesions (51, 52). Other metabolites of *CB*, such as vitamin B (VitB) and vitamin K (VitK), can also promote the growth of the body (39).

*FP* is a sort of highly oxygen-sensitive microorganism, which is classified as the Firmicutes and the main genus of the *Clostridium* (53). The metabolism derived from *FP* roughly includes butyrate, formate, and D-lactate (54). Moreover, it is one of the main components of the intestinal microbiome, and its amount accounts for a total bacteria above 5% (55). Another kind of Gram-positive bacterium, *Clostridium cocleatum*, acts in useful glucosidase activities, and its enzyme owns the function of decomposing the outermost potential barrier of mucin to implant in the gut (56). Other intestinal clostridia, such as *Clostridium nexile* and *Clostridium saccharogumia*, also originate from feces and are known to have certain beneficial effects.

Based on current evidence, some other *Clostridium* species (e.g., *Clostridium difficile*) may contribute to ADs by disrupting gut microbial homeostasis, secreting exotoxins, or exacerbating intestinal barrier dysfunction. This review focuses on the beneficial *Clostridium*, and the role of pathogenic strains in ADs warrants a separate, in-depth study.

## 3 The role of *Clostridium* as probiotics in ADs

**Figure 1:**
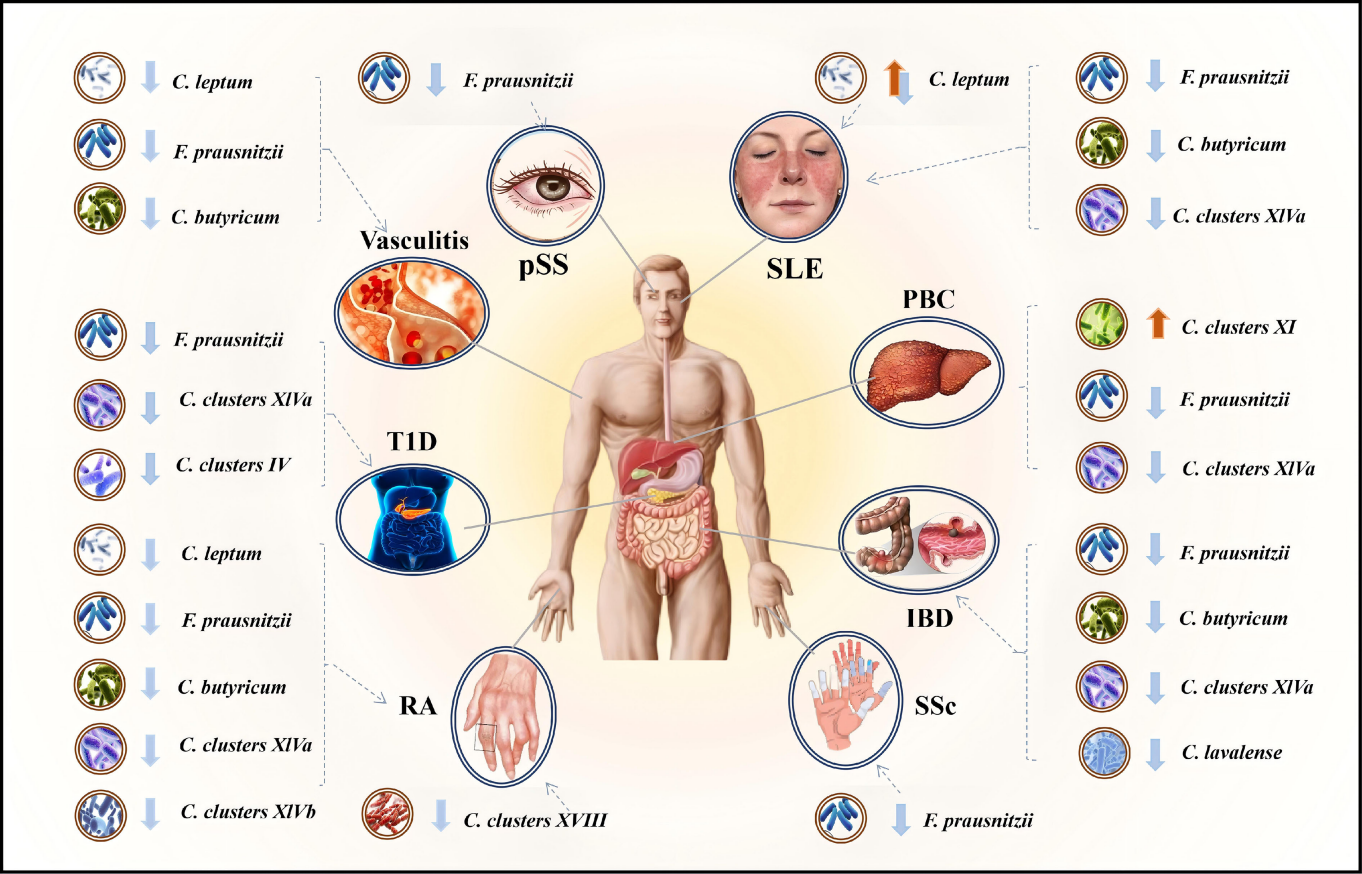
Alterations in abundance of beneficial *Clostridium* in autoimmune diseases. C., *Clostridium*; F., *Faecalibacterium*.

A growing body of research has revealed the unexpectedly beneficial roles of certain specific *Clostridium* species in the development and progression of ADs. These unique microbes not only modulate immunological homeostasis and inflammatory responses but also exert highly specialized protective effects in localized tissue environments. Now, we will mainly discuss changes in gut microbiota in ADs, as well as *Clostridium* and its metabolites that are responsive to ADs. As shown in **Figure 1**, alterations in the abundance of beneficial intestinal clostridia across different autoimmune diseases are presented.

### 3.1 Clostridium in IBD

IBD is an outcome of a combination of exogenous and endogenous pathogenic factors acting on the intestinal immune system (57, 58) and is broadly categorized as ulcerative colitis (UC) and Crohn’s disease (CD) (59). Its core pathogenesis involves intestinal mucosal barrier dysfunction, gut microbiota dysbiosis, and aberrant immune responses. IBD patients exhibit depleted beneficial bacteria (e.g., butyrate-producing *Clostridium*), increased pathogenic bacteria, and reduced SCFAs. This triggers broken immune tolerance: Overactivated innate immune cells secrete pro-inflammatory cytokines, CD4^+^ T cells skew toward pro-inflammatory subsets (T-helper 1 [Th1] cells/Th17), T regulatory cell (Treg) function is impaired, and autoantibodies exacerbate inflammation. These pro-inflammatory factors further damage the barriers and alter the microbiota, perpetuating the cycle of chronic inflammation (58, 59). Multiple clinical studies indicate the essential role of intestinal flora perturbations in the occurrence and development of IBD (60). There is a general decrease in taxonomic diversity in both CD and UC intestinal flora compared with healthy gut microbiomes (61), mainly characterized by phylum-level declines in *Bacteroidetes* and *Firmicutes*, and an upturn in *Proteobacteria* (62, 63). Specifically, this is mainly reflected in a reduction in the quantity and abundance of butyrate-producing bacteria, such as *CB*, *FP*, *Clostridium lavalense*, *Clostridium cluster XlVa*, *Blautia faecis*, *Bacteroides uniformis*, *Ruminococcus torques*, and *Roseburia inulinivorans* (64, 65).

Based on existing research, intestinal *Clostridium* and their metabolites exert effects on IBD mainly via intestinal barrier repair, innate immunity regulation, and adaptive immunity modulation (66–68). Notably, *Clostridium*-containing preparations also exhibit certain adjuvant therapeutic effects (69).

#### 3.1.1 Repair intestinal barrier and stabilize gut microbiota homeostasis

Intestinal barrier dysfunction initiates IBD. *Clostridium* and its metabolites act through “intestinal epithelial barrier repair” and “microbiota structure regulation,” supported by clinical and basic evidence (70–74). Clinical studies showed that post-colonoscopy *CB* supplementation can upregulate the expression of the buk gene (encoding butyrate kinase), enhance butyrate production, and accelerate homeostasis of the microbiota and colonic mucosal microenvironment (75). However, this study is limited by an extremely small sample size (four healthy individuals, seven *CB*-supplemented subjects).

Animal and *in vitro* experiments have further verified its barrier repair effect: Butyrate can promote proliferation and mucin secretion of intestinal epithelial cells (IPECs, Caco-2, HT-29), enhance the expression of tight-junction proteins (ZO-1, Claudin-1, Claudin-2), and maintain colonic mucosal integrity (76–82). *Clostridium* mixtures modulate microbiota composition (e.g., *Lactobacillus, Oscillospira*) to mitigate antibiotic-induced dysbiosis, reduce pathogenic bacterial translocation, and alleviate DSS-induced UC in mice (82, 83). *CB*-derived extracellular vesicles (EVs) decrease intestinal *Escherichia coli/Shigella* levels, enhance barrier integrity, and relieve UC symptoms (84–86). Hua et al. (87) found that *CB, when* combined with *Akkermansia muciniphila*, synergistically enhances barrier repair and increases sensitivity to anti-PD-L1 immunotherapy. Zhao et al. (79) showed that promoting *CB* proliferation and SCFA production repairs the colonic mucosal barrier in DSS-induced mice by upregulating tight-junction proteins and increasing goblet cell numbers, dependent on *CB*-mediated amino acid and 2-oxocarboxylic acid metabolism. Meanwhile, Wu et al. (78) demonstrated that butyrate repairs fecal microbiota dysbiosis *in vitro* and alleviates TNBS-induced colonic mucosal inflammation *in vivo*. Additionally, *FP* regulates intestinal epithelial NLRP6 inflammasome activity, promotes β-defensin-2/3 secretion, and competitively inhibits colonization by pro-inflammatory microorganisms such as *Candida albicans* and *Escherichia coli* (88).

#### 3.1.2 Inhibit innate immune overactivation via multiple signaling pathways

Beyond barrier repair, butyrate is a core anti-inflammatory mediator by regulating innate immunity (89–94). Clinical data have confirmed that butyrate levels are negatively correlated with colonic mucosal inflammation in IBD patients (77, 93). In the *in vitro* culture system of lamina propria mononuclear cells (LPMC) or peripheral blood mononuclear cells (PBMC) derived from CD patients, butyrate acts as a specific inhibitor of class I histone deacetylases (HDACI) and exhibits high responsiveness. Butyrate acts blocks the IL-6/STAT3/IL-17 and TLR2-MyD88-NF-κB pathways, and bidirectionally regulates cytokine secretion, Specifically, it upregulates anti-inflammatory factors such as IL-4 and IL-10 and downregulates pro-inflammatory factors such as CRP, IL-8, IL-12, and TNF-α (66, 67, 91, 92). Butyrate can also downregulate the expression of pro-inflammatory factors such as TNF-α by inhibiting NF-κB nuclear translocation and the degradation of inhibitor of nuclear factor κB alpha (IkBα), thereby exerting mucosal anti-inflammatory effects (93).

Meanwhile, butyrate also targets intestinal epithelial cells via EGFR-STAT3 and Nrf2 pathways. It enhances anti-inflammatory/antibacterial capacity in IPECs and upregulates Claudin-1, EGFR, and IL-10 gene expression (95). In Caco-2 intestinal epithelial cells, butyrate shows high responsiveness by activating the Nrf2 pathway. It forms a complementary regulatory network with the NLRP6 inflammasome, reduces ROS levels, and inhibits abnormal autophagy and the release of inflammatory factors (92). In addition, butyrate can activate the signaling pathway mediated by G protein-coupled receptors (GPRs), further inhibiting the downstream MEK-ERK and NF-κB pathways. This effect is not limited to specific cell types but can broadly block the pro-inflammatory activation of innate immune cells and amplify anti-inflammatory effect, as demonstrated (87, 93). Notably, *CB*’s anti-inflammatory effect exhibits gender heterogeneity in high-fat diet (HFD)-induced Fischer-344 rats (96). Males mainly restore fecal butyrate levels and inhibit the expression of TNF-α in the ascending colonic mucosa, whereas females focus on repairing intestinal epithelial tight-junction proteins (TJPs) and inhibiting myeloperoxidase (MPO) activity.

#### 3.1.3 Regulate innate immune cell function

Besides butyrate-mediated pathways, *Clostridium* (*FP*, *CB*) and their derivatives (extracellular matrix [EPM], EVs) directly regulate innate immune cells (97). Animal and *in vitro* experiments have clarified the cell type-specific regulatory mechanisms. At the level of innate lymphoid cells (ILCs), SCFAs stabilize hypoxia-inducible factor-1 (HIF-1), regulate the proportion of IL-22^+^ILC3 cells in the lamina propria of mice, and synchronously achieve intestinal barrier repair and inflammation reduction through the dual regulation of PI3K/AKT/mTOR and AMPK/mTOR pathways (79–81). The regulatory mechanism of dendritic cells (DC) is more diverse, as *FP* relies on its own extracellular matrix (EPM) to regulate cytokine balance in human monocyte-derived DC through the TLR2-dependent pathway, downregulating IL-12 and upregulating IL-10 (98). FP also induces IDO-1, PDL-1, CD39, and IL-27 in such DC through the TLR2/6-JNK pathway. It inhibits the release of pro-inflammatory factors in response to TLR4 stimulation and induces immune tolerance, a non-butyrate-dependent effect (99). *CB* activates the ERK-AP-1 pathway through TLR2, inducing TGF-β1 in mouse lamina propria DC (LPDCs) to promote iTreg generation (100). In macrophages, different *Clostridium* derivatives exert multifarious effects: *CB*-derived EVs inhibit the MAPK/NF-κB pathway through the miR-199a-3p-map3k4 axis, inducing M2 polarization (84–86). *FP*-derived EVs promote M2b polarization of human PBMC-derived macrophages, thereby inhibiting colonic fibrosis (101).

#### 3.1.4 Regulate Treg/Th17 balance and reconstruct immune tolerance

The imbalance of Treg/Th17 cells is central to the abnormal adaptive immunity in IBD. *Clostridium* and butyrate regulate this balance via multiple pathways, forming a multilevel immune tolerance network. Clinical studies have confirmed the immunomodulatory effects of CB in IBD. Yoshimatsu et al. (102) showed that *CB*-containing probiotics reduced recurrence rates (8.7% *vs*. 26.1%; 21.7% *vs*. 34.8%) by regulating Treg function and stabilizing the microbiota. Cai et al. (103) found that *CB* combined with specific immunotherapy (SIT) downregulated IL-6/IL-17, upregulated Breg, and enhanced Breg generation via butyrate-mediated HDAC1 inhibition. Both studies are limited by small sample sizes, short follow-up periods, and unclear parallel controls in the latter. *In vivo* and *in vitro* experiments clarify the hierarchical regulatory mechanisms: Butyrate directly regulates Th17/Treg homeostasis, promotes Treg differentiation through the HDAC1/FoxP3 pathway, and simultaneously inhibits Th17 activation and the expression of IL-17, IL-23, and RORγt (89, 90). Also, *CB* indirectly assists immune tolerance by upregulating retinol metabolism, reducing pro-inflammatory microbiota (77), and regulating intestinal permeability via miR-200c (80).

*Focus* on *FP: Clostridium* mixtures (*FP*, Bacteroides faecis, Roseburia intestinalis) restore the Treg/Th17 balance in DSS-induced UC mice, increase IL-10 and IL-4 secretion, reduce pro-inflammatory factors such as TNF, IL-1β, and IL-17, and repair the intestinal barrier (104). Sokol et al. (105) found that *FP* cell-free supernatant exerts non-butyrate-dependent anti-inflammatory effects by reducing colonic TNF-α/IL-12 and increasing serum IL-10. Sarrabayrouse et al. (106) demonstrated that *FP* induces IL-10^+^CD4^+^CD8αα^+^ (DP8α) Treg and upregulates FoxP3^+^Treg with specific recognition, whereas Touch et al. (107) confirmed DP8α Treg clones combined with *FP* alleviate colitis in humanized mice. Subsequent studies identified DC as the core target. Alameddine et al. (99) showed that *FP* induces DC expression of Treg-polarizing molecules (IDO-1, PD-L1, etc.) and inhibits TLR4-mediated secretion of pro-inflammatory factors. Rossi et al. (98) further showed that the FP extracellular matrix regulates the DC IL-10/IL-12 balance via a TLR2-dependent pathway, linking innate and adaptive immunity. Additionally, *Clostridium* sporogenes upregulate colonic IL-22 and promote Foxp3^+^Treg proliferation via metabolites (indole-3-propionic acid, etc.) in a non-butyrate-dependent manner (108). In summary, *Clostridium* repairs the Treg/Th17 balance through multiple mechanisms, offering new directions for IBD immunotherapy.

#### 3.1.5 Other mechanisms

*Clostridium*-containing fecal microbiota transplantation (FMT) and derivatives integrate the above mechanisms to treat IBD (109–111). Kashiwagi et al. (100) found that *CB* mixtures induce lamina propria DC to produce TGF-β1 via TLR2/ERK-AP-1, promoting iTreg generation and alleviating inflammation. FMT improves IBD model inflammation by regulating immunity, repairing mucosal barriers, stabilizing the microbiota (109, 110), and delivering *CB*-EVs to relieve UC (86). Clinical evidence showed that butyrate enhances conventional IBD drug efficacy. Vernia et al. (112) confirmed oral butyrate (4 g/day) combined with mesalazine (2.4 g/day) achieved a greater UCDAI score reduction (4.67 ± 2.19 *vs*. 2.54 ± 2.18) in mild-moderate UC patients via regulating intestinal epithelial energy supply and inhibiting inflammatory factors, with good safety.

Although current research on intestinal *Clostridium* and their metabolites in IBD intervention is extensive, it still has significant limitations. First, clinical evidence mostly relies on small-sample, short-follow-up single-center trials, and some preparations are mixed-strain, making it difficult to confirm the independent role of individual *Clostridium.* Second, there are substantial differences in gut microbiota between animal models and humans. Third, long-term safety data are scarce.

### 3.2 Clostridium in RA

RA is a kind of AD that affects the whole body and predominantly the joints (113), which is the result of immune dysregulation, such as aberrant immune activation and impaired immune tolerance (114). Differences in genetic makeup contribute to varied disease susceptibility among individuals, whereas environmental triggers initiate the pathological cascade. This process drives dysregulation of macrophage polarization and excessive production of pro-inflammatory cytokines and induces synovial fibroblast proliferation and activation. Concurrently, Th1/Th17 cells undergo aberrant activation, Tregs exhibit functional impairment, and B cells secrete autoantibodies, which form immune complexes that deposit in the synovium (115). More and more studies confirm that gut flora, such as *Bifidobacterium* and *Clostridium* (116, 117), plays a vital role in ameliorating immune dysfunction in RA (118).

Compared with healthy controls (HCs), RA patients exhibit significant differences in gut microbial load, community richness, and diversity (119). Namely, 37 bacterial species are enriched and 21 are depleted in RA patients (120). *Actinomyces* (121, 122), *Prevotella* (123), *Bacteroides* (120, 124), *Escherichia–Shigella* (125) are the main species increased, whereas *Mycobacterium*, *Lactobacillus*, *Enterobacter* (123), *Deltaproteobacteria*, and *Clostridiaceae* decreased (126), including *CB*, *FP*, *Clostridium leptum*, *Clostridium cluster XlVa*, *Clostridium cluster XlVb*, *Clostridium cluster XVIII* (127). Meanwhile, Picchianti et al. (126) used a clinical cohort of 42 RA patients and 10 healthy controls found that RA patients exhibit clear gut microbiota dysbiosis, and the *Clostridium* metabolite vitamin K2 (VitK2) was enriched. Unlike findings from most studies, Gomez et al. (128) found that *Clostridium* is more abundant in the gut of arthritis-susceptible mice. The core discrepancies may stem from two factors, first, distinct genetic backgrounds of mouse strains, with inherent differences in gut microbiota responses to disease between arthritis-susceptible and conventional models, and second, undefined *Clostridium* strains, which fail to rule out the possibility of pathogenic subtypes.

*Clostridium* and its metabolites directly modulate the core immune dysregulation in RA (129). Dysregulated macrophage polarization is pivotal in RA pathogenesis. Overactivated M1 macrophages exacerbate inflammation through secreting pro-inflammatory cytokines, driving joint damage, whereas immunosuppressive M2 macrophages are diminished, disrupting immune equilibrium (130). Song et al. (131) demonstrated in animal models and clinical studies that CB modulates macrophage polarization (M1→M2) via butyrate. This occurs through miR-146a upregulation, which targets and suppresses the SOCS7/JAK2-STAT3 pathway, thereby inhibiting pro-inflammatory cytokines and promoting an anti-inflammatory microenvironment. Additional research confirmed that miR-146a synchronously promoted M2 polarization by inhibiting pro-inflammatory mediators TRAF6 and IRAK-1, while suppressing the TLR2-associated MyD88/NF-κB pathway to reduce inflammation (132, 133). These findings provide a theoretical basis for exploring the role of miR-146a in RA macrophage polarization.

In the study of *Clostridium*’s role in regulating adaptive immunity, Furusawa et al. (134) used a germ-free mouse model colonized with *Clostridium* and an *in vitro* induction experiment of CD4^+^T cells. Using NMR metabolomics and flow cytometry to detect the Treg proportion, it was found that butyrate, as a histone deacetylase (HDAC) inhibitor, can enhance histone H3 acetylation of the Foxp3 gene and directly promote the differentiation of colonic Tregs. He et al. (135) used a quasi-paired clinical cohort of 29 RA patients and a collagen-induced arthritis (CIA) mouse model. Through metagenomic sequencing and *in vitro* coculture experiments of peripheral blood mononuclear cells (PBMCs), it was verified that butyrate can selectively expand peripheral blood Tregs in RA patients, inhibit the activation of conventional T cells (Tconvs), downregulate the production of anti-citrullinated peptide antibodies (ACPA), and reduce cartilage and bone tissue damage. Moreover, Takahashi et al. (136) adopted a CIA mouse model and an *in vitro* iTFR cell culture system. Via high-butyrate diet intervention and adoptive transfer experiments, it confirmed that a high-butyrate diet can induce the differentiation of follicular Tregs through HDAC inhibition, suppress the production of type II collagen (CII)-specific autoantibodies, and reduce joint swelling and bone erosion. Another study using the CIA mouse model also suggested that *Clostridium cluster XIVa* can activate transforming growth factor-β (TGF-β), thereby enhancing the immunosuppressive function of Tregs (125). Meanwhile, this strain can induce IL-10 secretion by secreting polysaccharide A (PSA), synergistically promote Treg proliferation, reduce the levels of pro-inflammatory cytokines such as TNF-α and IL-6, and thus inhibit the systemic inflammatory response in RA. Based on the above research findings, we hypothesize that beneficial *Clostridium* and its metabolites regulate adaptive immunity and induce immune tolerance in RA, thereby synergistically alleviating joint immune damage. However, the key limitations of existing studies lie in small clinical sample sizes without multicenter validation and unstandardized intervention protocols in animal models, both of which impede clinical translation.

Clostridial products have also been found to exert joint-protective and other adjunctive immunomodulatory effects. He et al. (135) also reported that the butyrate levels in RA patients correlate negatively with RANKL expression, a marker of osteoclast activation. *In vitro* experiments further confirmed that butyrate directly inhibits osteoclast differentiation and reduces joint bone erosion. A standardized meta-analysis approach by Volkova et al. (137) found that FP was widely recognized as a helpful strain for modulating immune homeostasis, with a positive correlation with B vitamins. It was also shown to exert detrimental effects on certain cholesterol esters and acylcarnitines. Moreover, the enzymes produced by *Clostridium* can facilitate the conversion of glucocorticoids into androgens, which are involved in immunomodulation (138).

Despite the promising findings regarding the role of *Clostridium* and its metabolites in RA, several key issues remain to be addressed, including insufficient clinical evidence and unclear strain specificity.

### 3.3 Clostridium in SLE

SLE (lupus), commonly known as lupus, is a disease characterized by aberrant immune system activity (139). Its core pathogenesis lies in the synergistic interaction between genetic susceptibility (e.g., gene variants) and environmental triggers (e.g., ultraviolet radiation and gut microbiota dysbiosis), leading to multiple immune system disorders. On the one hand, dendritic cells in the innate immune system overproduce type I interferons. On the other hand, in the adaptive immune system, Tregs exhibit functional defects, whereas Th17 and Tfh cells undergo abnormal activation. These T cells further assist B cells in producing large amounts of autoantibodies, leading to the formation of immune complexes that deposit in multiple organs. These complexes activate the complement system and mediate inflammatory damage, ultimately resulting in multisystem involvement affecting the skin, kidneys, joints, and other organs (140). Similar to other ADs, the gut microbiota also play an important role in the development of SLE, and their relative abundance can be affected by SLE (141).

In clinical studies, various taxa of gut microbiota in patients with SLE exhibit significant and distinct changes in abundance. A lower *Firmicutes/Bacteroidetes* ratio (142), reduced counts of *Lactobacillaceae* (143), and altered abundance of several genera have been reported (144) including *Eggerthella*, *Klebsiella*, *Flavonifractor*, *Lachnospiraceae* (145, 146), *Prevotella*, *Eubacterium, and Rhodococcus* were significantly enriched, whereas *Pseudobutyrivibrio* and *Dialister* were decreased in SLE patients (147). Importantly, Firmicutes, a phylum that includes *Clostridium*, exhibits a negative correlation with the SLE disease activity index (SLEDAI) (148, 149). In most studies, the abundance of various beneficial *Clostridium* has declined dramatically, including *CB*, *FP*, *Clostridium cluster XIVa*, and *Clostridium leptum* (145, 147). Nevertheless, in some clinical studies, contradictory findings have been reported. *Clostridium leptum* (150) and *Clostridium cluster XI* (151) were found to be enriched in SLE gut microbiota and reduced after treatment. During disease remission, the restoration of gut microenvironment homeostasis leads to demand-dependent reductions in the abundance of *Clostridium leptum* and *Clostridium cluster XI*, classified as potential “conditional beneficial bacteria,” which align with the principle of dynamic gut microbial balance and may not be directly linked to therapeutic efficacy.

SCFAs, as the primary metabolic products of beneficial *Clostridium*, can exert various beneficial effects on different stages of SLE (152). In the early stage of disease, SCFAs have protective effects in SLE by suppressing pathobiont (153). In TLR7-dependent lupus mice, SCFAs inhibit the abundance and intestinal translocation of the pathobiont *Lactobacillus reuteri*. This suppression indirectly reduces the recruitment of plasmacytoid dendritic cells (pDCs) and the activation of interferon pathways (153). As the disease progresses, butyrate modulates gut microbiota to protect against target organ damage, particularly in the context of environmental trigger-induced dysbiosis. In the MRL/lpr lupus mouse model, butyrate supplementation reverses SLE-related gut microbiota dysbiosis and protects against renal damage (154). Specifically, butyrate increases the abundance of beneficial taxa, including the Firmicutes phylum, Clostridia class, Lachnospiraceae family, and *Clostridium* leptum, while reducing the proportion of the Bacteroidetes phylum. This microbiota modulation is accompanied by significant amelioration of renal pathology, supporting the role of butyrate in regulating the gut–immune–kidney axis to protect SLE target organs (154).

Moreover, SCFAs, particularly butyrate, exert multifaceted regulatory roles in correcting immune dysregulation (155). The animal experiment results demonstrated that butyrate plays a key immunomodulatory role in SLE by targeting B cells through epigenetic mechanisms (156, 157). As a histone deacetylase (HDAC) inhibitor, butyrate acts directly on B-cell intrinsic pathways without relying on GPR signaling or serving as an energy substrate. Specifically, it upregulates specific microRNAs (miRNAs) to silence the expression of two critical molecules: miR-155, miR-181b, and miR-361 target Aicda, whereas miR-23b, miR-30a, and miR-125b target Prdm1 (encoding B lymphocyte-induced maturation protein-1, Blimp-1). This epigenetic silencing effectively inhibits class-switch DNA recombination (CSR), somatic hypermutation (SHM), and plasma cell differentiation, thereby reducing the production of both T-dependent and T-independent autoantibodies (e.g., anti-dsDNA antibodies). In the MRL/Fas(lpr/lpr) lupus mouse model, this mechanism significantly ameliorates disease manifestations and extends survival (157). However, these *in vitro* and animal studies lack validation in human SLE patients, and the optimal butyrate concentration for clinical application remains undetermined.

*Clostridium* itself also contributes to immune tolerance restoration in SLE. *Clostridium clusters IV* and *XIVa* are key inducers of colonic Tregs and play a pivotal role in restoring immune tolerance in SLE. Atarashi et al. (158) demonstrated that colonization of germ-free (GF) mice with a cocktail of 46 *Clostridium* (primarily belonging to *clusters IV* and *XIVa*) robustly increases Foxp3+ Treg numbers in the colonic lamina propria (LP) without affecting small intestinal Tregs. Notably, this Treg induction is independent of Toll-like receptor (TLR)/Nucleotide-binding Oligomerization Domain (NOD)/Card9 pattern recognition receptor signaling pathways. The induced Tregs are predominantly the IL-10^+^cytotoxic-T-lymphocyte-associated antigen-4 (CTLA4) ^high^ Helios-subset with potent immunosuppressive activity, and they can migrate to extraintestinal organs to suppress systemic inflammation.

Clinical evidence further validates the therapeutic potential of the *Clostridium*–butyrate axis in SLE. A randomized (159), double-blind, placebo-controlled trial involving 46 adult SLE patients (23 in the synbiotic group, 23 in the placebo group) showed that 60 days of synbiotic supplementation significantly increased butyrate metabolism, reduced the pro-inflammatory cytokine IL-6, and inhibited the elevation of hs-CRP. Importantly, the SLE Disease Activity Index 2K (SLEDAI-2K) score in the synbiotic group improved dramatically from 14 (9, 16) to 8 (2, 12) (p<0.001), whereas no significant change was observed in the placebo group. Additionally, synbiotic supplementation improved gut microbiota functional imbalance (159). Limitations of this trial include a small sample size and unclear contributions of specific strains in the synbiotic formulation.

In addition to immune and microbiota regulation, butyrate also exerts auxiliary metabolic effects that may indirectly benefit SLE patients. It can enhance energy production capacity by regulating nutrient intake and promoting fat oxidation through sensitization of brown adipose tissue (160). This metabolic regulatory function may synergize with caloric restriction, which is a strategy proven to impede the development of lupus-like disease in New Zealand Black (NZB) and NZB×New Zealand White (NZW) F1 mice (161, 162).

### 3.4 Clostridium in T1D

T1D is a classic kind of AD (163). The pathogenesis of T1D in the majority of cases is synergistically precipitated by genetic and environmental factors, leading to a pancreatic β-cell-specific autoimmune attack involving multiple immune cell types and molecules. These pathogenic processes ultimately result in massive β-cell loss and absolute insulin deficiency, thereby inducing dysregulation of glucose homeostasis (164). Numerous studies have demonstrated the impact of the intestinal flora on the autoimmune response and disease outcomes (165).

According to the exploration results of the T1D patients or models, the following bacteria were mainly increased in abundance: *Bifidobacterium longum* (166, 167), *Eubacterium rectale* (168), *Bacteroides uniformis* (169), *Lachnospira* spp., *Intestinimonas* spp., *Micrococcales* spp (170)., *Actinomyces* spp (171)., *Bacteroides* spp (172)., *Prevotella* spp (168)., *Ruminococcus* spp (173)., and *Veillonella* spp (174)., whereas the bacterium with decreased abundance mainly includes butyrate-producing bacterium, lactic acid-producing bacterium, and mucin-degrading bacterium, such as *Lactococcus*, *Lactobacillus (*173), *Streptococcus* (174), *Bifidobacterium*, *Proteobacteria* (172), *Subdoligranulum*, *Roseburia*, *Eubacterium*, *Anaerostipes* (175), *FP* (176, 177), *Clostridium clusters IV* (178, 179), and *XIVa* (180, 181). Notably, the depletion of *Clostridium clusters IV/XIVa* and butyrate-producing taxa is closely linked to impaired gut barrier function and β-cell autoimmunity, laying the foundation for the core regulatory role of *Clostridium* and butyrate in T1D.

The regulation of β-cells is the core mechanism controlling the initiation and progression of T1D. To clarify the protective effects of *Clostridium* and its metabolites on β-cells, researchers have conducted a series of targeted studies. In T1D, elevated intestinal mucosal permeability exacerbates antigen translocation, and these translocated antigens can directly target pancreatic β-cells, amplifying aberrant autoimmunity (182). However, maintaining gut barrier integrity relies on adequate butyrate production (183). Through a randomized, double-blind, placebo-controlled trial involving 20 pediatric T1D patients, Reddi et al. (184) put forward that β-cell autoimmunity correlates with reduced abundance of butyrate- and lactate-producing bacteria. Meanwhile, in a nonobese diabetic (NOD) mice model, Tanca et al. (185) demonstrated that expression of butyrate biosynthetic enzymes was observably decreased in NOD mice.

Emerging evidence from *in vitro* and *in vivo* studies confirms that butyrate exerts direct protective effects on pancreatic β-cells through multiple pathways. Specifically, in secretion-competent INS-1E cells and isolated mouse islets (186), butyrate significantly attenuates IL-1β- or IL-1β/IFN-γ-induced apoptosis by suppressing endoplasmic reticulum (ER) stress, while reducing the production of nitric oxide (NO) and chemokines (CXCL1, CXCL10) that amplify immune cell infiltration. Complementary studies in mouse islets (187) further demonstrate that butyrate alleviates IL-1β-induced mitochondrial dysfunction. Butyrate promotes mitochondrial hyperfusion (via upregulating fusion proteins Opa1/Mfn2 and downregulating fission protein Fis1), enhances glucose-stimulated oxygen consumption and mitochondrial membrane potential, and restores redox homeostasis by increasing reduced glutathione (GSH) levels and the GSH/GSSG ratio. These functional protective effects are consistent with metaproteomic findings in children with recent-onset T1D (188), where gut microbiota dysregulation (including reduced butyrate-producing taxa) correlates with β-cell damage severity, supporting a clinical link between butyrate deficiency and T1D pathogenesis.

Beyond direct β-cell protection, butyrate exerts indirect protective effects by modulating immune responses and promoting the differentiation of Tregs that shield pancreatic β-cells from cytokine-induced damage (189, 190). Mechanistic studies in mouse islets and INS-1E cells (191) further clarify that butyrate inhibits IL-1β-induced expression of inflammatory genes (Nos2, Cxcl1, and Ptgs2) and NO production by suppressing NF-κB activation. Based on these comprehensive research findings, we speculate that the regulation of the T1D immune system and inflammatory pathways may be closely linked to butyrate, a key metabolite produced by *Clostridium*.

Besides the core mechanism mediated by butyrate, other SCFAs and beneficial *Clostridium* also exhibit certain immunomodulatory effects. On the one hand, other SCFAs produced by *Clostridium* exert immunomodulatory effects in T1D by reducing intestinal permeability (192). Intestinal translocated LPS to the portal vein is known to contribute to obesity-induced low-grade inflammation and insulin resistance in mice (193), whereas SCFAs counteract these pathogenic processes. In NOD Myd88^−/−^mice, a SCFA-rich diet increased butyrate and acetate levels in feces, hepatic tissue, and peripheral blood, thereby protecting against T1D (194, 195). In NOD mice, SCFA supplementation further reduced islet-autoreactive T cells; promoted Foxp3^+^ Treg proliferation in pancreatic lymph nodes, the spleen, and the gut mucosa; and enhanced immune tolerance (196, 197). Similarly, T1D incidence decreased significantly in NOD mice treated with a prebiotic diet supplemented with SCFAs (198). Clinically, Lo Conte et al. (199) documented gut barrier damage with mucus layer alterations in T1D patients, which correlated with reduced abundance of SCFA-producing bacteria (e.g., *CB*). This cross-sectional study, however, is limited by a small sample size and the inability to confirm causality between SCFA depletion and gut barrier impairment.

On the other hand, *Clostridium* itself can also exert a multi-pathway immunomodulatory effect to alleviate T1D. Recently, Jia et al. (200) made a hypothesis that supplementation with the well-characterized probiotics *CB* CGMCC0313.1 (*CB*0313.1) might regulate and control the pancreatic Tregs and prevent diabetes in NOD mice. The results showed that early oral administration of *CB*0313.1 mitigated insulitis, delayed the onset of diabetes, and improved energy metabolic dysfunction. The protective effects may involve increased Tregs, rebalanced Th1/Th2/Th17 cells, and a less proinflammatory immunological milieu in the gut, peripheral lymph nodes (pLN), and pancreas. An increase of α4β7^+^ Tregs in the pLN suggested enhanced migration of gut-primed Tregs to the pancreas. Furthermore, 16S rRNA sequencing revealed that *CB*0313.1 upregulated the *Firmicutes/Bacteroidetes* ratio, enriched *Clostridium*, and promoted butyrate-producing taxa.

### 3.5 *Clostridium* in primary Sjögren’s syndrome

The primary Sjögren’s syndrome (pSS) is a type of AD characterized by inflammation and destruction of exocrine glands, primarily manifesting as xerophthalmia and xerostomia (201). Its core pathogenesis involves genetic susceptibility combined with environmental triggers, which activate antigen-presenting cells and initiate abnormal immune responses. Lymphocyte infiltration of exocrine glands, autoantibody production, and an imbalance in the inflammatory cytokine network drive disease progression, ultimately resulting in glandular dysfunction and multisystem involvement. Systemic manifestations may include arthritis, interstitial lung disease (ILD), renal and nervous system involvement, and an increased risk of lymphoma (202).

Using human and mouse models, the researchers have verified that gut flora changes in pSS. The flora with increased abundance includes *Lactobacillus salivarius*, *Staphylococcus*, *Corynebacterium*, *Bacteroides fragilis*, *Ruminococcus gnavus*, *Veillonella parvula*, *Pseudobutyrivibrio*, *Escherichia/Shigella*, and *Streptococcus parasanguinis* (201, 203, 204). On the contrary, *Bacteroides*, *Parabacteroides*, *FP*, *Clostridium leptum*, and *Prevotella* were significantly decreased, and there was a 50% reduction in the high butyrate producer *FP* (205).

Numerous studies have reported that intestinal *Clostridium* can ameliorate pSS-related disorders and associated severe immune-inflammatory responses. Accumulating evidence further demonstrates that in pSS models, reduced gut microbiome diversity and diminished production of microbiota-derived metabolites lead to decreased tolerogenic dendritic cells (DCs) and Th17/Treg imbalance, consequently promoting autoantibody accumulation and exacerbation of glandular inflammatory responses in pSS (203). At this juncture, beneficial *Clostridium* metabolites exert multiple therapeutic effects in pSS. Animal model studies have preliminarily revealed that *Clostridium* colonization can curtail the overgrowth of pro-inflammatory microbiota, restore the intestinal mucosal barrier, and limit systemic translocation of bacterial LPS, ultimately reestablishing gut microbial homeostasis (206, 207). This series of effects is of great significance due to its relevance to the pathogenic origins of pSS. Concurrently, a clinical study involving 98 pSS patients and 105 healthy controls (208) found that butyrate-producing bacteria were significantly reduced in pSS patients, and this reduction correlated negatively with increased Th17 cells and an imbalance in Th17/Treg. These bacteria can correct the imbalance between Treg/Th17 and macrophage M1/M2 via butyrate production, thereby reducing glandular inflammation. However, this cross-sectional clinical study cannot definitively establish a causal relationship between reduced bacterial levels and immune imbalance. Additionally, in pSS-related experimental animal studies, SCFAs modulate aberrant activation of B cells and dendritic cells by activating the aryl hydrocarbon receptor (AhR) or by suppressing NF-κB signaling, thereby alleviating lymphocyte infiltration in exocrine glands (209).

However, existing research still faces issues such as insufficient reliability in demonstrating clinical causal relationships, overreliance on animal models, and inadequate elucidation of underlying mechanisms. In the future, it is necessary to establish animal models.

### 3.6 *Clostridium* in vasculitis

Vasculitides, mainly including Kawasaki disease (KD) and Henoch–Schönlein purpura (HSP), are initiated by abnormal autoimmune function. Through inflammatory cell infiltration (neutrophils, T/B cells), cytokine network imbalance, and vascular wall structural destruction, it ultimately leads to vascular stenosis, occlusion, or rupture, resulting in tissue and organ ischemia or hemorrhage (210, 211). In this kind of ADs, beneficial *Clostridium* and their metabolites also exhibit considerable therapeutic potential.

Some clinical studies demonstrate variations in *Clostridium* abundance in patients with vasculitis. Fabi et al. (212) enrolled 13 KD, 10 HSP, and 12 non-KD febrile illness children to investigate intestinal flora alterations in these ADs. They found that the intestinal flora differed significantly from that of controls. Moreover, this study identified potential KD- and HSP-specific characteristics, including reduced abundances of *Dialister* in KD patients and *Clostridium* and *Akkermansia* in HSP patients. In the research conducted by Wang et al. (213), the transformation of intestinal flora, especially the decrease of SCFAs producing bacteria, was demonstrated in the KD mouse model.

To elucidate the therapeutic role of *Clostridium* in vasculitis, researchers established vasculitis animal models and used the probiotic *CB* and antibiotic cocktails to modulate intestinal flora. The results showed that administration of *CB* significantly increased the abundance of SCFA-producing bacteria, relieved arterial lesions, and decreased the levels of inflammatory markers IL-1β and IL-6, whereas antibiotics depleted intestinal flora and exacerbated the inflammatory response (213).

Several other studies have further demonstrated the beneficial effects of SCFAs produced by *Clostridium*. The SCAFs may exert regulatory effects upstream of the pathogenesis of vasculitis while also exerting multifaceted anti-inflammatory effects. *In vitro*, butyrate increased the expression of mitogen-activated protein kinase phosphatase-1 (MKP-1), which dephosphorylates activated c-Jun N-terminal kinase (JNK), ERK1/2, and p38 Mitogen-activated protein kinase (MAPK) to counteract excessive inflammation in RAW264.7 macrophages (214). Experimental findings in mouse KD models demonstrate that reduced abundance of specific *Clostridium* (e.g., *Clostridium sensu stricto*, *Clostridium clusters XIVa*) correlates significantly with vasculitis progression, whereas supplementation with these *Clostridium* or SCFAs alleviates vascular inflammation by either activating G protein-coupled receptors (GPR43/109A) or inhibiting HDAC activity. This process thereby downregulates the NF-κB pathway, augments Treg differentiation, and suppresses Th17 expansion and inflammatory cytokine release (215, 216).

Current studies on beneficial *Clostridium* in vasculitides have obvious shortcomings. The few existing clinical studies are limited by small sample sizes, and most are merely cross-sectional studies. While supplementation with *Clostridium* has shown therapeutic effects on vasculitis animal models, its efficacy in patients remains to be confirmed. Meanwhile, animal experiments have focused only on KD mice, lacking models related to HSP.

### 3.7 Clostridium in PBC

PBC is a chronic autoimmune liver disease featuring immune-mediated destruction of intrahepatic bile ducts. The classic pathogenic pathway can be initiated by endogenous environmental disturbances such as intestinal dysbiosis. This is followed by multiple immune abnormalities: marked impairment of the immunosuppressive function of myeloid-derived suppressor cells (MDSCs), pro-inflammatory polarization of Kupffer cells, T-cell differentiation imbalance, and B-cell hyperactivation that induces antimitochondrial antibody (AMA)-mediated cytotoxicity. These factors collectively drive the progressive damage of biliary epithelial cells by autoantibodies, leading to cholestasis, fibrosis, and potential progression to cirrhosis or liver failure (217). Many patients also exhibit gut dysbiosis, characterized by decreased *FP* (218).

Beneficial *Clostridium* may exert beneficial effects on the core pathogenesis of PBC. Clinical studies by Wang et al. demonstrated that butyrate can enhance the immunosuppressive function of myeloid-derived suppressor cells (MDSCs) in patients with PBC (219). Furthermore, using a murine cholangitis model, the researchers clarified the underlying mechanisms: butyrate activates peroxisome proliferator-activated receptor delta (PPARD) to promote fatty acid β-oxidation (FAO)-mediated metabolic reprogramming; it also triggers an HDAC3-mediated epigenetic mechanism that enhances acetylation of lysine 27 on histone H3 (H3K27ac). These two pathways synergistically induce the expansion of MDSCs and augment their immunosuppressive activity, thereby exerting anti-inflammatory and immunomodulatory effects and providing a novel therapeutic strategy for PBC treatment. However, this study has notable clinical limitations: The second cohort (Cohort 2) only included 24 patients, confounding factors such as diet and concurrent medications were not adjusted for, and there is a lack of clinically standardized protocols for MDSC function detection.

In PBC livers, Kupffer cells polarize to the pro-inflammatory M1 phenotype, which is positively correlated with hepatic inflammation severity (220). Fu et al. (221) performed a multi-tiered study (including clinical, *in vivo* and *in vitro* research) to investigate how PCS inhibits Kupffer cell immune response and excessive inflammation. PCS could promote the polarization of Kupffer cells from M1 to M2, reducing the inflammatory response. In murine models, PCS treatment reduced pro-inflammatory factors (IL-1β, IL-6, CCL3, and TNF-α) and significantly increased the anti-inflammatory factor IL-10, thereby further alleviating hepatic inflammation. Thus, PCS supplementation via diet or direct administration may serve as a potential therapeutic strategy for PBC. Nevertheless, the study fails to clarify differences in serum PCS levels among PBC patients at different disease stages, uses non-human primary Kupffer cells *in vitro*, and does not evaluate the safety threshold for clinical PCS supplementation, which limits its therapeutic translation.

Furthermore, several animal model studies have suggested that certain gut Clostridia species may alleviate hepatic inflammation by modulating bile acid metabolism through their influence on immune-related pathways, such as the Farnesoid X receptor (FXR)/Vascular adhesion protein-1 (VAP-1) and the 7α-dehydroxylation reaction (222–224). However, these studies lack supporting human clinical data, and the specific molecular mechanisms underlying bile acid metabolism regulation remain to be validated.

Current studies share some limitations. For example, preclinical models and animal-derived cells fail to recapitulate human PBC pathology and metabolic features.

### 3.8 *Clostridium* in scleroderma

As an immune-mediated rheumatic disease, SSc, or scleroderma, causes fibrosis of the skin and internal organs as well as vascular disease (225). Its core pathogenesis involves the interplay of three key processes: immune dysregulation that is characterized by Treg dysfunction and B-cell hyperactivation, vascular endothelial injury and dysfunction, and abnormal fibroblast proliferation (226). These jointly lead to systemic multi-tissue fibrosis and progressive organ dysfunction.

Some researchers have identified common changes in gut flora in SSc. Concretely, the beneficial commensal genera decreased, including *FP* (226, 227), *Clostridiaceae*, *Rikenella*, *Bacteroides*, *Prevotella*, *Roseburia*, *Pseudobutyrivibrio*, *Escherichia/Shigella*, and *Raecalibacterium* (228), whereas the pathobiont genera rose, including *Lactobacillus* (229), *Ruminococcus*, *Akkermansia*, *Bifidobacterium*, *Fusobacterium*, *Erwinia*, and *Streptococcus* (230, 231).

Zhang et al. (216) utilized C57BL/6J mice to induce a model of SSc, confirming that mesenchymal stem cells (MSCs) alleviate SSc symptoms by modulating the balance of the gut microbiota. Specifically, this effect is mediated through an increase in butyrate-producing bacteria (such as *FP*, *Roseburia*, *Butyricicoccus porcorum*, and *Gemmiger formicilis*) and a decrease in conditionally pathogenic bacteria (including *Akkermansia* and *Parasutterella excrementihominis*). These alterations reduce systemic inflammation, promote immune tolerance, and decrease autoimmune attacks, thereby contributing to the relief of SSc symptoms. In several clinical studies, alterations in FP emerged as a noteworthy observation; specifically, it was found to be depleted in patients with SSc (232). The therapeutic role of *Clostridium* in SSc may still depend on the regulation of Th17 by *FP*, as well as on Tregs (232).

Research on the association between SSc and beneficial *Clostridium* remains scarce. Current evidence is limited to small-sample clinical observations and animal models, with no large-scale randomized controlled trials (RCTs).

The pathogenesis of each autoimmune disease and the therapeutic targets of beneficial intestinal *Clostridium* are illustrated in **Table 2**; **Figures 2**–**9**.

**Table 2:** Therapeutic role of beneficial *Clostridium* in autoimmune diseases.

Disease	Gut Clostridia species	Primary effector molecules	Primary regulatory targets	Primary health outcomes	Experimental model	Reference IDs
Inflammatory bowel disease	*CB*	Probiotic formulation	Upregulates buk gene, enhances butyrate production	Accelerates microbiota and colonic mucosal homeostasis	Clinical study	(75)
	Butyrate	Butyrate	1. Promotes proliferation and mucin secretion of IECs 2. Enhances tight-junction proteins (ZO-1, Claudin-1, Claudin-2)	Maintains colonic mucosal integrity	*In vitro* experiment (IPECs, Caco-2, HT-29)	(76–82)
	*Clostridium* mixture (containing *CB*)	Probiotic formulation	Modulates microbiota composition	1. Mitigates antibiotic-induced dysbiosis 2. Alleviates DSS-induced colitis	Animal experiment	(82, 83)
	*CB*	EVs	1. Reduces *E. coli/Shigella* levels 2. Enhances barrier integrity	Relieves UC symptoms	Animal experiment	(84–86)
	*CB*	Combined bacterial preparation	Synergistic barrier repair	Enhances anti-PD-L1 immunotherapy sensitivity	Animal experiment	(87)
	*CB*	Butyrate	Upregulates tight-junction proteins via CB-mediated amino acid and 2-oxocarboxylic acid metabolism	Repairs colonic mucosal barrier in DSS-induced mice	Animal experiment	(79)
	Butyrate	Butyrate	1. Repairs fecal microbiota dysbiosis 2. Alleviates TNBS-induced inflammation	Alleviates colonic mucosal inflammation	*In vitro* + animal experiment	(78)
	*FP*	Bacterial cellular metabolites	1. Regulates NLRP6 inflammasome 2. Promotes β-defensin-2/3 secretion	Competitively inhibits colonization of *C. albicans* and *E. coli*	*In vitro* experiment + animal experiment	(88)
	Butyrate	Butyrate	1. HDACI inhibitor 2. Blocks IL-6/STAT3/IL-17 and TLR2-MyD88-NF-κB pathways	1. Upregulates IL-4, IL-10 2. Downregulates CRP, IL-8, IL-12, TNF-α	*In vitro* (LPMC/PBMC from CD patients)	(66, 67, 91, 92)
	Butyrate	Butyrate	1. Inhibits NF-κB nuclear translocation 2. Inhibits IkBα degradation	Downregulates TNF-α; exerts mucosal anti-inflammatory effects	*In vitro*/animal models	(93)
	Butyrate	Butyrate	Activates EGFR-STAT3 and Nrf2 pathways in IECs	1. Upregulates Claudin-1, EGFR, IL-10 2. Enhances anti-inflammatory capacity	*In vitro* experiment (IPECs)	(95)
	Butyrate	Butyrate	1. Activates Nrf2 2. Complements NLRP6 regulation 3. Reduces ROS	Inhibits abnormal autophagy and inflammatory factor release	*In vitro* experiment (Caco-2)	(92)
	Butyrate	Butyrate	1. Activates GPRs 2. Inhibits downstream MEK-ERK & NF-κB	Broadly blocks pro-inflammatory activation of innate immune cells	*In vitro* + animal experiment	(87, 93)
	*CB*	Butyrate	Gender-heterogeneous effects	1. Inhibits TNF-α (M) 2. Inhibits MPO activity (F)	Animal experiment	(96)
	SCFAs (from *Clostridium*)	SCFAs	1. Stabilizes HIF-1 2. Regulates PI3K/AKT/mTOR and AMPK/mTOR	1. Regulates IL-22^+^ILC3 proportion 2. Repairs barrier 3. Reduces inflammation	Animal experiment	(79–81)
	*FP*	EPM	TLR2-dependent pathway in human monocyte-derived DCs	1. Downregulates IL-12 2. Upregulates IL-10	*In vitro* experiment	(98)
	*FP*	Bacterial cell-free supernatant	1. Induces IDO-1, PD-L1, CD39, IL-27 via TLR2/6-JNK 2. Inhibits TLR4 signaling	1. Inhibits pro-inflammatory factors 2. Induces immune tolerance	*In vitro* experiment	(99)
	*CB* mixture	Probiotic formulation	Activates ERK-AP-1 via TLR2, inducing TGF-β1 in LPDCs	Promotes iTreg generation	Animal experiment	(100)
	*CB*	EVs	Inhibits MAPK/NF-κB via miR-199a-3p-map3k4 axis	Induces M2 macrophage polarization	*In vitro* experiment	(84–86)
	*FP*	EVs	Modulates macrophage metabolism	Promotes M2b polarization; inhibits colonic fibrosis	*In vitro* experiment	(101)
	*CB*	Probiotic formulation	Regulates Treg function and stabilizes microbiota	Reduces UC recurrence rates	Clinical study	(102)
	*CB*	Butyrate	HDACI inhibition enhances Breg generation	1. Downregulates IL-6/IL-17 2. Upregulates Bregs 3. Ameliorates UC	Clinical study	(103)
	Butyrate	Butyrate	1. Promotes Treg differentiation via HDAC1/FoxP3 2. Inhibits Th17/IL-17/RORγt	Regulates Th17/Treg homeostasis	Animal/*in vitro* experiment	(89, 90)
	*CB*	Butyrate	1. Upregulates retinol metabolism 2. Regulates intestinal permeability via miR-200c	1. Reduces pro-inflammatory microbiota 2. Assists immune tolerance	Animal experiment	(77, 80)
	*Clostridium* mixture (containing *FP*)	Bacterial cellular metabolites	Restores Treg/Th17 balance	1. Increases IL-10, IL-4 2. Reduces TNF, IL-1β, IL-17 3. Repairs intestinal barrier	Animal experiment	(104)
	*FP*	Bacterial cell-free supernatant	Exerts anti-inflammatory effects	1. Reduces colonic TNF-α/IL-12 2. Increases serum IL-10	Animal experiment	(105)
	*FP*	Bacterial immunomodulatory properties	1. Induces IL-10^+^CD4^+^CD8αα^+^ (DP8α) Tregs 2. Upregulates FoxP3^+^Tregs	Suppresses intestinal inflammation	Animal experiment + *In vitro*/humanized mice	(106, 107)
	*Clostridium* sporogenes	Bacterial metabolites	1. Upregulates colonic IL-22 2. Promotes Foxp3^+^Treg proliferation	Protects against colonic inflammation	Animal experiment	(108)
	FMT	Integrated mechanisms	1. Regulates immunity 2. Repairs barriers 3. Stabilizes microbiota	Improves IBD inflammation	Animal experiment + clinical study	(86, 109, 110)
	Butyrate	Butyrate	1. Regulates intestinal epithelial energy supply 2. Inhibits inflammatory factors	Achieves greater UCDAI score reduction in mild-moderate UC	Clinical study	(112)
Rheumatoid arthritis	*CB*	Butyrate	miR-146a upregulation inhibits SOCS7/JAK2–STAT3 pathway	1. IL-6 and TNF-α expression downregulated 2. Macrophage polarization from M1 to M2 phenotype induced	Clinical study + animal experiment	(131)
	*Clostridium*	Butyrate	1. miR-146a mediates TRAF6 and IRAK-1 inhibition 2. TLR2–MyD88–NF-κB signaling cascade blocked	1. Pro-inflammatory mediators suppressed 2. Joint inflammation mitigated	Animal experiment + *in vitro* experiment	(132, 133)
	Butyrate-producing *Clostridium*	Butyrate	1. FoxP3 histone H3 acetylation induced 2. HDAC inhibited	1. Treg differentiation promoted 2. Mucosal barrier function reinforced	Animal experiment + *in vitro* experiment	(134)
	*Clostridium*	Butyrate	1. Tfh cell function modulated 2. Treg function enhanced 3. HDAC inhibition expands peripheral Tregs, downregulates ACPA	1. Autoantibody production reduced 2. Immune homeostasis restored 3. Cartilage and bone damage reduced	Animal experiment + clinical study	(135)
	*Clostridium*	Butyrate	HDAC inhibitory activity mediates downstream immune regulation	1. Anti-collagen II autoantibodies inhibited 2. Symptoms improved	Animal experiment	(136)
	*Clostridium cluster XIVa*	Bacterial cellular metabolites	Activates TGF-β	Enhances the immunosuppressive function of Tregs	Animal experiment	(125)
	*Clostridium cluster XIVa*	PSA	Induces IL-10 secretion	1. Synergistically promotes Treg proliferation 2. Reduces pro-inflammatory cytokines (TNF-α, IL-6)	Animal experiment	(127)
	*Clostridium*	VitK2	Correlates with RA-enriched bacteria	Clinical association observed	Clinical study	(126)
	*FP*	Bacterial cellular metabolites	1. Associated with B vitamin metabolism 2. Modulates cholesterol ester and acylcarnitine metabolism	Systemic immune homeostasis regulated	Clinical study (meta-analysis)	(137)
	*Clostridium* scindens	Bacterial metabolic enzymes	Glucocorticoid-to-androgen conversion regulated	Immunomodulation achieved	*In vitro* experiment	(138)
Systemic lupus erythematosus	*Clostridium*	Butyrate	1. HDAC modulation regulates B-cell epigenetic activity 2. T cell-independent immune response modulated	Autoimmune responses inhibited to contribute to systemic immune regulation	Animal experiment	(153–155)
	*Clostridium*	SCFAs	Systemic immunomodulation achieved	1. Albuminuria reduced to reflect improved renal function 2. Splenomegaly suppressed to indicate systemic inflammation alleviation	Animal experiment	(156)
	*Clostridium*	Butyrate	1. Brown adipose tissue oxidation enhanced 2. Energy metabolism regulated	Caloric restriction–mimicking effects delay lupus progression	Animal experiment	(157–159)
	*Clostridium*	Butyrate	Intestinal epithelial barrier strengthened	1. LPS and LPS-producing bacterial translocation blocked 2. Renal pathological damage alleviated	Animal experiment	(160)
	Synbiotic (containing *Clostridium*)	Synbiotic formulation	Increases butyrate metabolism	1. Reduces pro-inflammatory cytokine IL-6 and inhibits hs-CRP elevation 2. Significantly improves SLEDAI-2K score 3. Improves gut microbiota functional imbalance	Clinical study (RCT)	(159)
	*Clostridium cluster IV/XIVa*	Bacterial cellular metabolites	Treg accumulation promoted to contribute to immune tolerance	1. SLE disease activity negatively regulated 2. Treg deficiency compensated to correct immune dysregulation	Animal experiment + clinical study	(152, 161, 162)
	*Clostridium cluster XIVa*	Bacterial immunomodulatory properties	Lamina propria Treg subset differentiation induced to enhance local immune regulation	Immune tolerance balance reestablished to prevent aberrant activation	Animal experiment	(163)
Type 1 diabetes	*Clostridium*	Butyrate	1. ER stress markers (CHOP, p-eIF2α, ATF4) inhibited to alleviate cellular stress 2. Pro-apoptotic genes (DP5, PUMA) downregulated to reduce apoptosis	Pancreatic β-cells protected from aberrant apoptosis to maintain endocrine function	*In vitro* experiment + animal experiment	(185–187)
	*Clostridium*	Butyrate	1. HDAC inhibition enhances H3K27ac modification 2. Mitochondrial function and redox homeostasis improved to support cellular resilience	β-cell functional integrity maintained to support glucose homeostasis	*In vitro* experiment + animal experiment	(187)
	*Clostridium*	Butyrate	1. Treg differentiation promoted to enhance regulatory immune activity 2. NF-κB inhibited to reduce IL-1β and nitric oxide production	β-cells protected from cytokine-induced damage to prevent diabetes progression	*In vitro* experiment + animal experiment	(188)
	Butyrate-/lactate-producing bacteria	Bacterial cellular metabolites	Abundance correlates with β-cell autoimmunity	β-Cell autoimmunity correlates with reduced abundance	Clinical study (RCT)	(184)
	Butyrate-producing *Clostridium*	Bacterial metabolic enzymes	Expression of butyrate biosynthetic enzymes decreased	Associated with disease state in NOD mice	Animal experiment	(185)
	*Clostridium*	SCFAs	Intestinal permeability reduced to preserve barrier integrity	LPS translocation blocked to attenuate insulin resistance	Animal experiment	(192, 193)
	*Clostridium*	SCFAs	Peripheral blood butyrate and acetate enriched to strengthen mucosal immunity	1. Diabetes onset delayed 2. Autoreactive T cells reduced to mitigate autoimmune damage	Animal experiment	(194, 195)
	*Clostridium*	SCFAs	1. FoxP3^+^ Treg increased in pancreatic lymph nodes and spleen to promote systemic tolerance 2. Gut mucosal immunity modulated to improve host–microbe interactions	1. Islet autoimmunity suppressed 2. Immune tolerance enhanced	Animal experiment	(196)
	SCFAs-producing *Clostridium*	Bacterial cellular metabolites	Gut microbiota abundance modulated to restore microbial balance	Mucus barrier integrity maintained	Clinical study	(198)
	*CB*0313.1	Probiotic formulation	1. Treg expression enhanced to strengthen immune regulation 2. Th1/Th2/Th17 cell balance restored to re-establish immune homeostasis	1. Diabetes onset delayed to reduce early disease risk 2. Insulitis attenuated to protect pancreatic islets 3. Energy metabolic dysfunction improved to support systemic balance 4. Gut microbial homeostasis reestablished	Animal experiment	(200)
Primary Sjögren’s syndrome	*Clostridium*	SCFAs	1.tDCsmodulated to promote immune tolerance 2. Th17/Treg balance corrected to restore immunological equilibrium	1. Autoantibody accumulation reduced to limit autoimmune damage 2. Glandular inflammation alleviated to improve exocrine gland function	Animal experiment	(202, 206)
	*Clostridium*	Bacterial colonization and ecological competition	1. Pro-inflammatory microbiota overgrowth suppressed to limit dysbiosis 2. Intestinal mucosal barrier integrity restored to maintain gut health	1. Systemic LPS translocation blocked to prevent systemic inflammation 2. Gut microbial homeostasis reestablished to enhance host–microbe interactions	Animal experiment	(206, 207)
	*Clostridium*	Bacterial cellular metabolites	1.Correct the balance of Treg/Th17 2. Macrophage M1/M2 polarization balanced to regulate pro- and anti-inflammatory responses	Immune cell balance restored to prevent excessive activation	Clinical study	(208)
	*Clostridium*	Propionate/butyrate	1. AhR activated to support anti-inflammatory signaling 2. NF-κB signaling inhibited to reduce inflammatory activity	1. Aberrant B-cell and dendritic cell activation suppressed to improve tolerance 2. Lymphocyte infiltration in exocrine glands reduced to decrease tissue injury	Animal experiment	(209)
Vasculitis	*CB*	Probiotic formulation	1. Gut barrier function maintained to preserve host defense 2. Microbial composition modulated to reinforce intestinal stability	1. Intestinal inflammation reduced to alleviate mucosal damage 2. SCFA-producing bacterial abundance increased to support anti-inflammatory effects 3. Coronary lesions attenuated to protect cardiovascular health	Animal experiment	(213)
	*CB*	Butyrate	MKP-1 phosphatase upregulated to induce JNK/ERK1/2/p38 MAPK dephosphorylation	Macrophage hyperinflammation suppressed to alleviate immune overactivation	*In vitro* experiment	(214)
	*CB*	Butyrate/acetate /propionate	1. GPR43 and GPR109A receptors activated to mediate anti-inflammatory effects 2. HDAC activity inhibited to regulate epigenetic pathways	1. NF-κB signaling suppressed to reduce inflammatory activity 2. Treg differentiation promoted to enhance anti-inflammatory effects	Animal experiment + *in vitro* experiment	(215, 216)
Primary biliary cholangitis	*Clostridium*	Butyrate	1. PPARD activated to promote FAO 2. HDAC3-mediated H3K27ac modification regulates metabolic signaling	1. MDSCs expanded to enhance immunosuppressive capacity 2. Hepatic inflammation reduced to improve liver health	Clinical study + animal experiment	(219)
	*Clostridium*	PCS	Kupffer cell polarization from M1 to M2 phenotype promoted to support hepatic immune regulation	1. Pro-inflammatory factors (IL-1β, IL-6, TNF-α) decreased to suppress inflammation 2. IL-10 increased to alleviate intrahepatic bile duct injury	Animal experiment + *in vitro* experiment	(220)
	*Clostridium*	Bacterial metabolic enzymes	Bile acid metabolism modulated to contribute to systemic metabolic regulation	1. Systemic inflammation reduced 2. Immune tolerance established 3. Autoimmune attacks diminished	Animal experiment	(222–224)
Systemic sclerosis	Butyrate-producing *Clostridium*	Butyrate	MSC-mediated microbiota modulation supports host–microbe balance	1. Systemic inflammation reduced to lower overall disease activity 2. Immune tolerance established to prevent recurrent autoimmunity 3. Autoimmune attacks diminished to improve long-term outcomes	Animal experiment	(226–232)
	*FP*	Bacterial cellular metabolites	1. Th17/Treg balance regulated to maintain immune homeostasis 2. Immune cell differentiation modulated to enhance systemic immunoregulation	Cutaneous and visceral fibrosis alleviated to improve tissue integrity	Clinical study + Animal experiment	(232)

*CB*, *Clostridium butyricum*; SCFAs, short-chain fatty acids; HDAC1, histone deacetylase 1; FoxP3, forkhead box P3; GPRs, G protein-coupled receptors; NF-κB, nuclear factor kappa B; IL, interleukin; STAT3, signal transducer and activator of transcription 3; CRP, C-reactive protein; Th17, T helper 17 cell; Treg, regulatory T cell; RALDH2, retinaldehyde dehydrogenase 2; IgA, immunoglobulin A; HIF-1, hypoxia-inducible factor 1; PI3K, phosphoinositide 3-kinase; AKT, protein kinase B; mTOR, mammalian target of rapamycin; ILC3, innate lymphoid cell type 3; NLRP6, NOD-like receptor family pyrin domain containing 6; Nrf2, nuclear factor erythroid 2-related factor 2; *FP*, *Faecalibacterium prausnitzii*; TNF-α, tumor necrosis factor alpha; IgE, immunoglobulin E; Breg, B regulatory cell; IDO-1, indoleamine 2;3-dioxygenase 1; PD-L1, programmed death-ligand 1; CD39, cluster of differentiation 39; TLR4, Toll-like receptor 4; DP8α, DP8 alpha T cell; TLR2, Toll-like receptor 2; ERK, extracellular signal-regulated kinase; AP-1, activator protein 1; TGF-β1, transforming growth factor beta 1; DSS, dextran sulfate sodium; miR-146a, microRNA-146a; SOCS7, suppressor of cytokine signaling 7; JAK2, Janus kinase 2; TRAF6, TNF receptor-associated factor 6; IRAK-1, interleukin-1 receptor-associated kinase 1; MyD88, myeloid differentiation primary response 88; Tfh, follicular helper T cell; ACPA, anti-citrullinated peptide antibody; ER, endoplasmic reticulum; CHOP, C/EBP homologous protein; eIF2α, eukaryotic translation initiation factor 2 alpha; ATF4, activating transcription factor 4; PUMA, p53 upregulated modulator of apoptosis; H3K27ac, histone H3 lysine 27 acetylation; LPS, lipopolysaccharide; NO, nitric oxide; Th1, T helper 1 cell; Th2, T helper 2 cell; tDCs, tolerogenic dendritic cells; M1, macrophage type 1; M2, macrophage type 2; AhR, aryl hydrocarbon receptor; GPR43, G protein-coupled receptor 43; GPR109A, G protein-coupled receptor 109A; MKP-1, mitogen-activated protein kinase phosphatase-1; JNK, c-Jun N-terminal kinase; MAPK, mitogen-activated protein kinase; PPARD, peroxisome proliferator-activated receptor delta; FAO, fatty acid β-oxidation; HDAC3, histone deacetylase 3; MDSCs, myeloid-derived suppressor cells; PCS, p-cresol sulfate; FXR, farnesoid X receptor; VAP-1, vascular adhesion protein-1; MSC, mesenchymal stem cell; EVs, extracellular vesicles; EPM, extracellular polymeric matrix; FMT, fecal microbiota transplantation.

**Figure 2:**
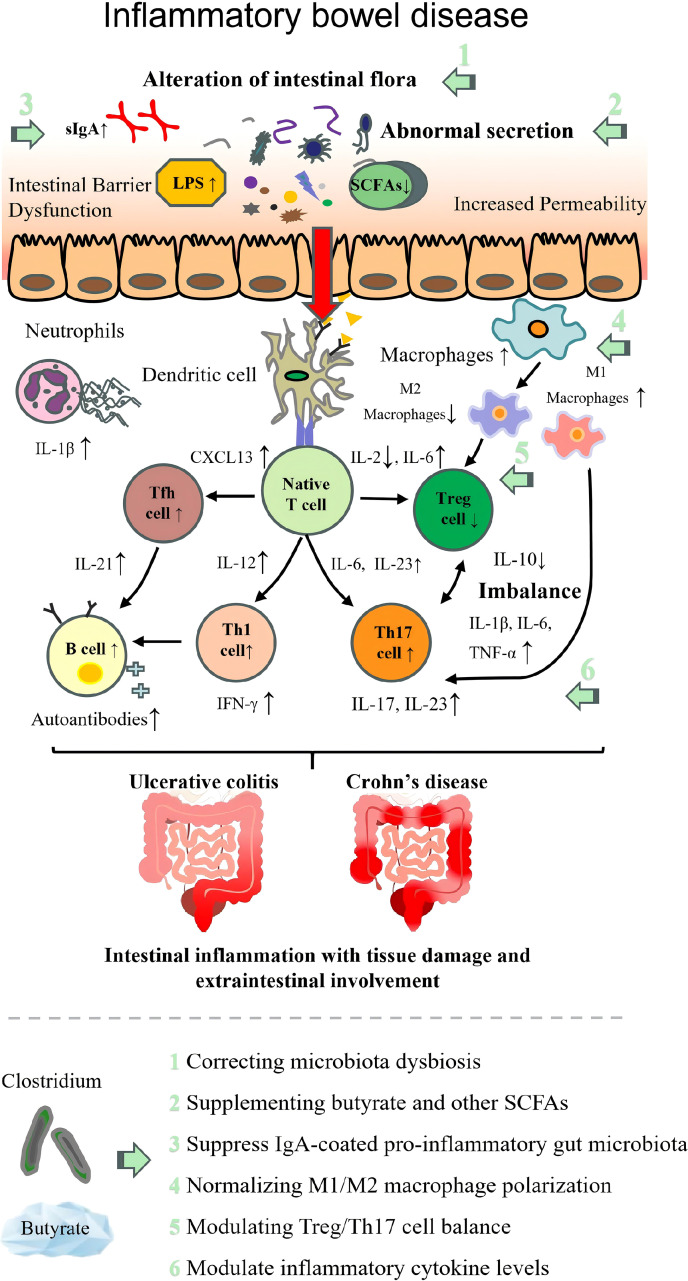
Pathogenesis of inflammatory bowel disease with therapeutic targets of beneficial *Clostridium*. LPS, lipopolysaccharide; SCFAs, short-chain fatty acids; Th17, T helper 17 cell; Treg, regulatory T cell; Tfh cell, follicular helper T cell; IL, interleukin; TNF-α, tumor necrosis factor α; IFN-γ, interferon γ.

**Figure 3:**
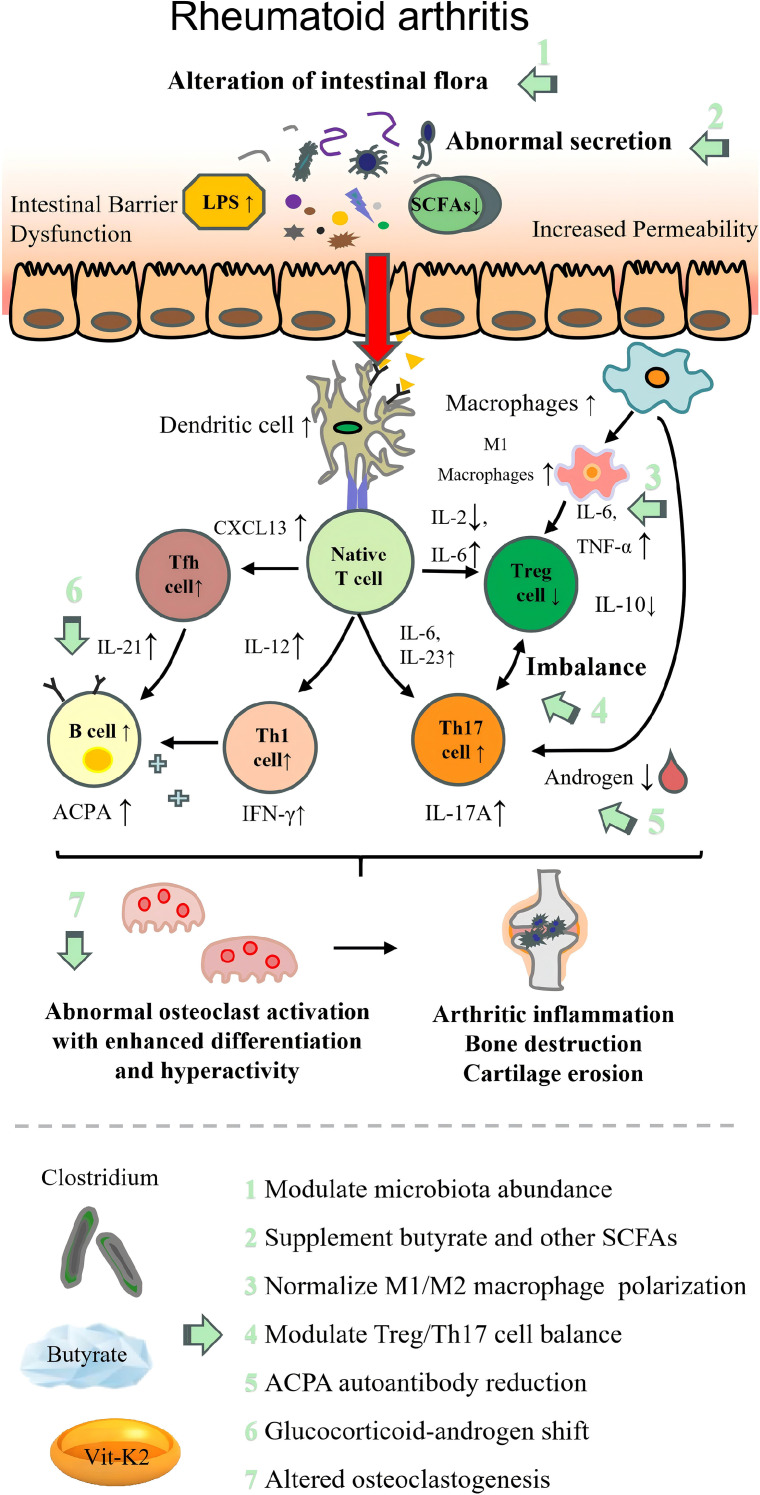
Pathogenesis of rheumatoid arthritis with therapeutic targets of beneficial *Clostridium*. LPS, lipopolysaccharide; SCFAs, short-chain fatty acids; Th17, T helper 17 cell; Treg, regulatory T cell; Tfh cell, follicular helper T cell; IL, interleukin; TNF-α, tumor necrosis factor α; IFN-γ, interferon γ; ACPA, anti-citrullinated peptide antibodies; CXCL3, C–X–C motif chemokine ligand 3; VitK2, vitamin K2.

**Figure 4:**
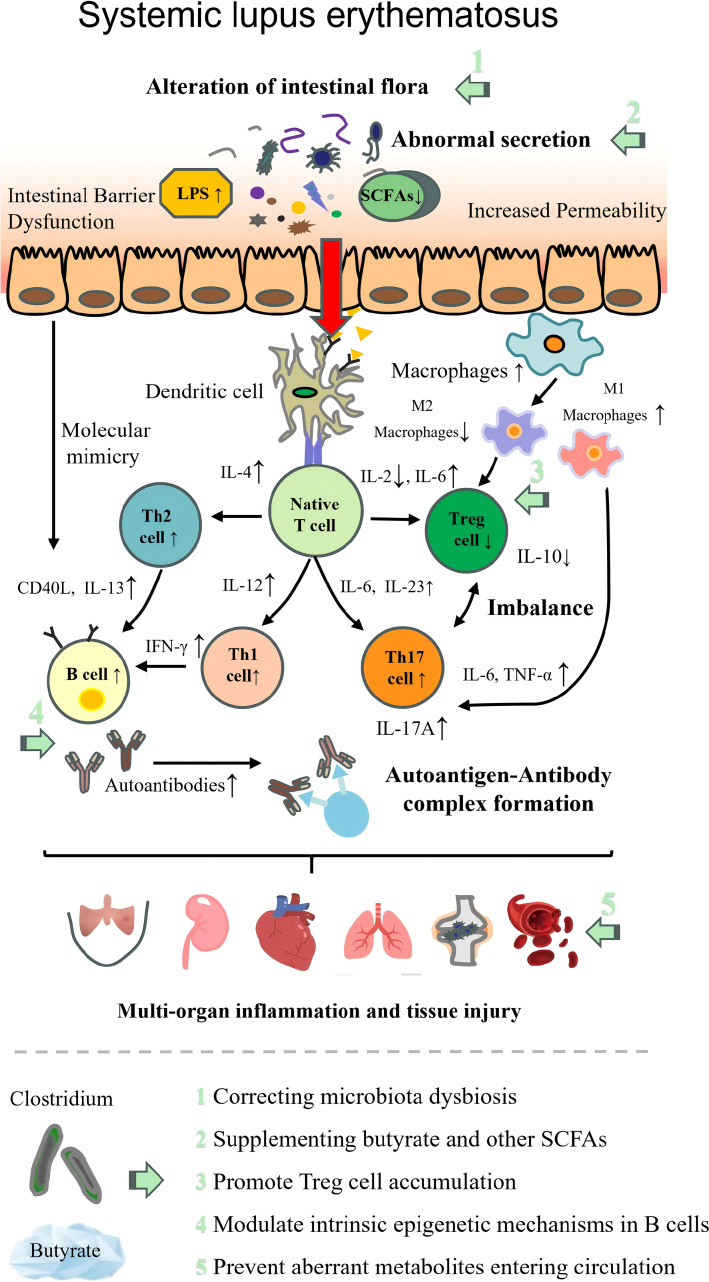
Pathogenesis of systemic lupus erythematosus with therapeutic targets of beneficial *Clostridium*. LPS, lipopolysaccharide; SCFAs, short-chain fatty acids; Th1, T helper 1 cell; Th2, T helper 2 cell; Th17, T helper 17 cell; Treg, regulatory T cell; IL, interleukin; TNF-α, tumor necrosis factor α; IFN-γ, interferon γ; CD40L, CD40 ligand.

**Figure 5:**
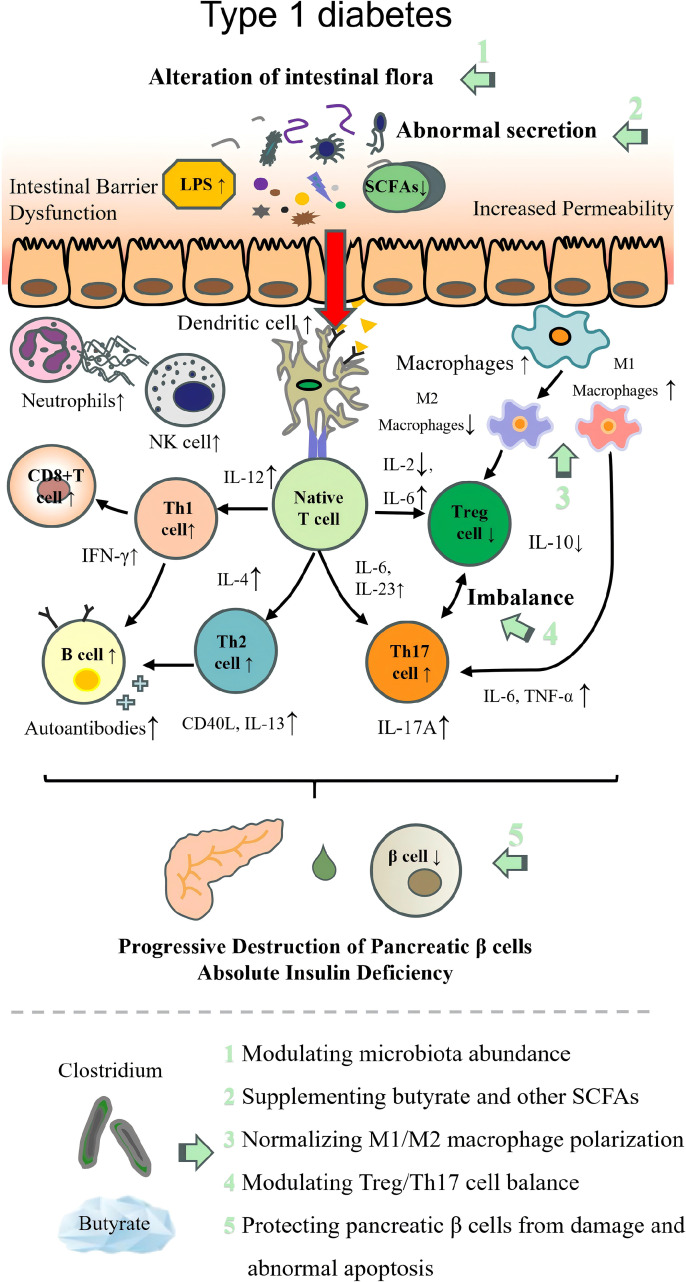
Pathogenesis of type 1 diabetes with therapeutic targets of beneficial *Clostridium*. LPS, lipopolysaccharide; SCFAs, short-chain fatty acids; Th1, T helper 1 cell; Th2, T helper 2 cell; Th17, T helper 17 cell; Treg, regulatory T cell; NK cell, natural killer cell; IL, interleukin; TNF-α, tumor necrosis factor α; IFN-γ, interferon γ.

**Figure 6:**
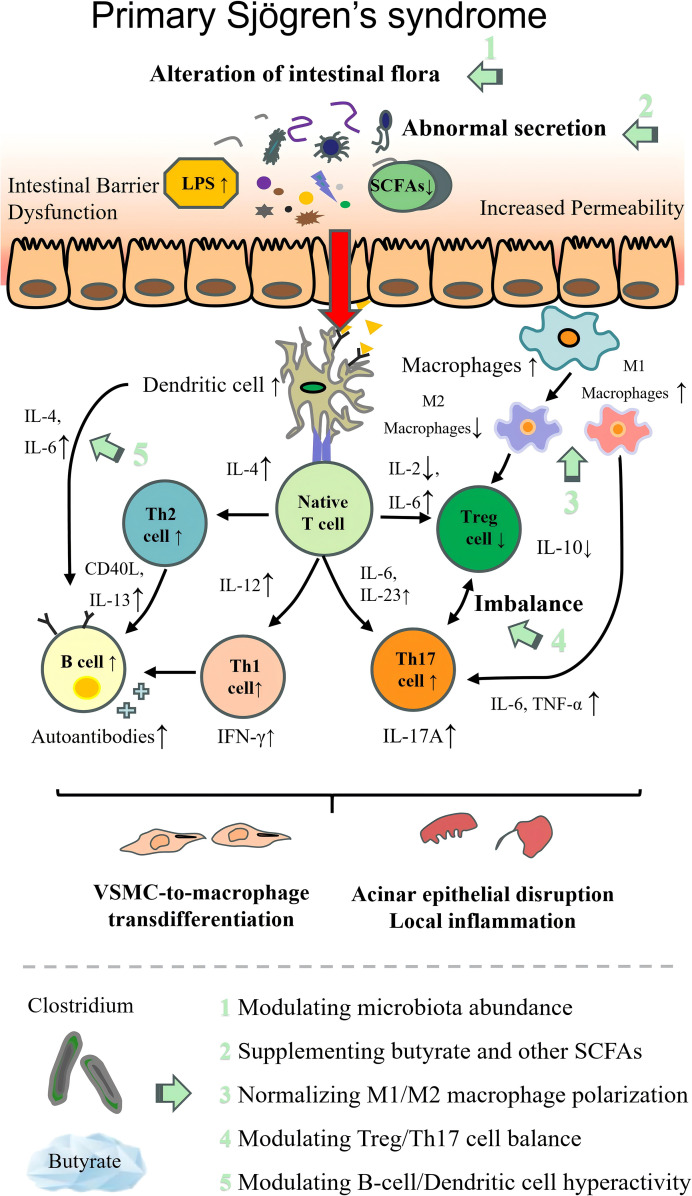
Pathogenesis of primary Sjögren’s syndrome with therapeutic targets of beneficial *Clostridium*. LPS, lipopolysaccharide; SBA, secondary bile acids; Th1 cell, T helper 1 cell; Th2 cell, T helper 2 cell; Th17 cell, T helper 17 cell; Treg cell, regulatory T cell; IL, interleukin; TNF-α, tumor necrosis factor α; IFN-γ, interferon γ; CD40L, CD40 ligand.

**Figure 7:**
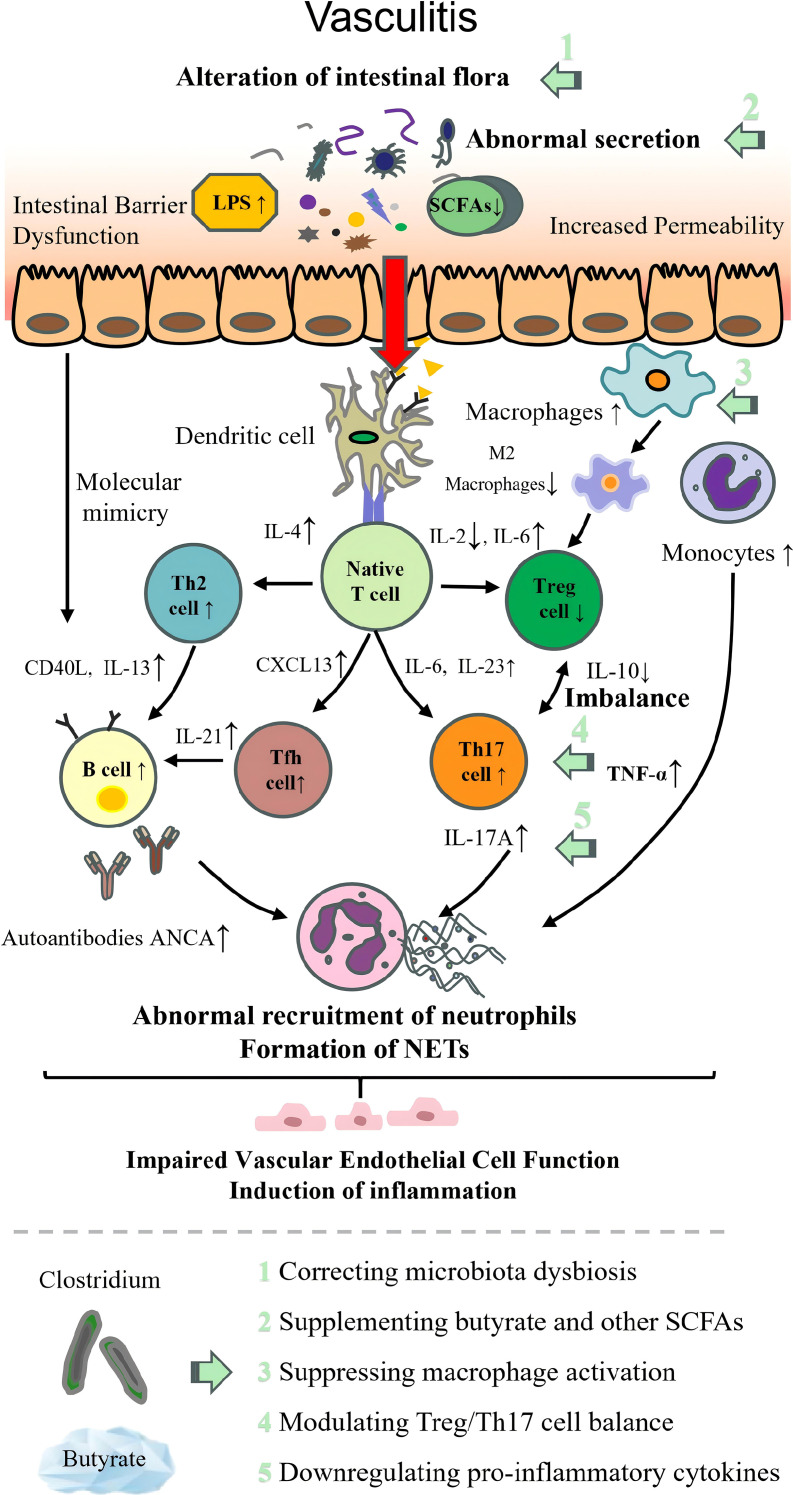
Pathogenesis of vasculitis with therapeutic targets of beneficial *Clostridium*. LPS, lipopolysaccharide; SCFAs, short-chain fatty acids; Tfh cell, follicular helper T cell; Treg, regulatory T cell; Th1, T helper 1 cell; Th2, T helper 2 cell; Th17, T helper 17 cell; IL, interleukin; TNF-α, tumor necrosis factor α; CD40L, CD40 ligand; CXCL13, C–X–C motif chemokine ligand 13; ANCA, anti-neutrophil cytoplasmic antibody; NETs, neutrophil extracellular traps.

**Figure 8:**
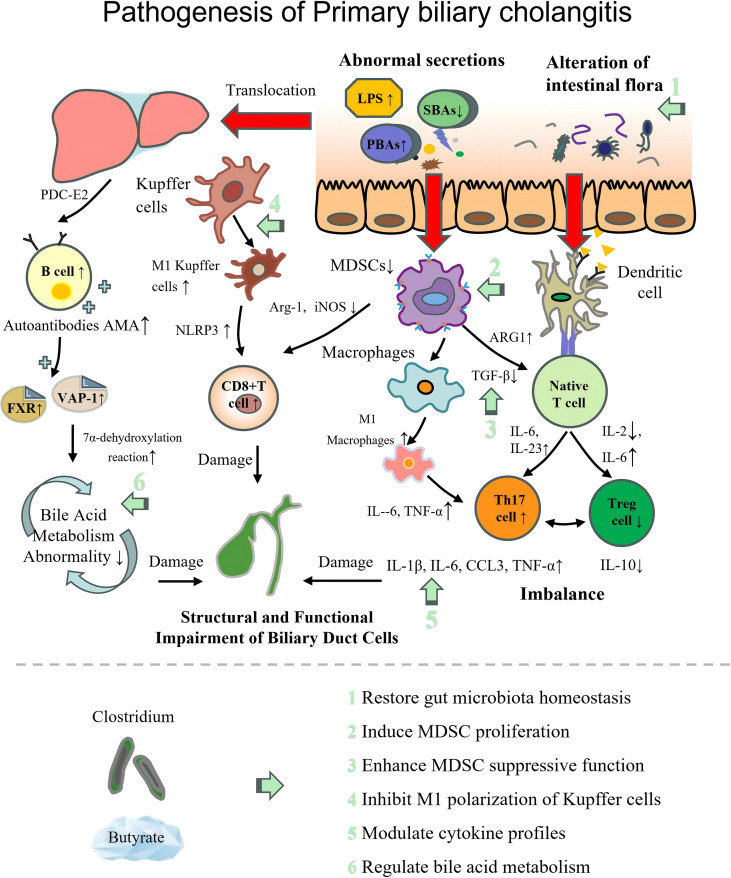
Pathogenesis of primary biliary cholangitis with therapeutic targets of beneficial *Clostridium*. LPS, lipopolysaccharide; SBA, secondary bile acids; PBA, primary bile acids; Th1 cell, T helper 1 cell; Th2 cell, T helper 2 cell; Th17 cell, T helper 17 cell; Treg cell, regulatory T cell; MDSCs, myeloid-derived suppressor cells; IL, interleukin; TNF-α, tumor necrosis factor α; AMA, anti-mitochondrial antibodies; NLRP3, NLR family pyrin domain containing 3; PDC-E2, pyruvate dehydrogenase complex-E2; ARG1, arginase 1; iNOS, inducible nitric oxide synthase; TGF-β, transforming growth factor β; CCL3, C–C motif chemokine ligand 3; FXR, farnesoid X receptor; VAP-1, vascular adhesion protein-1.

**Figure 9:**
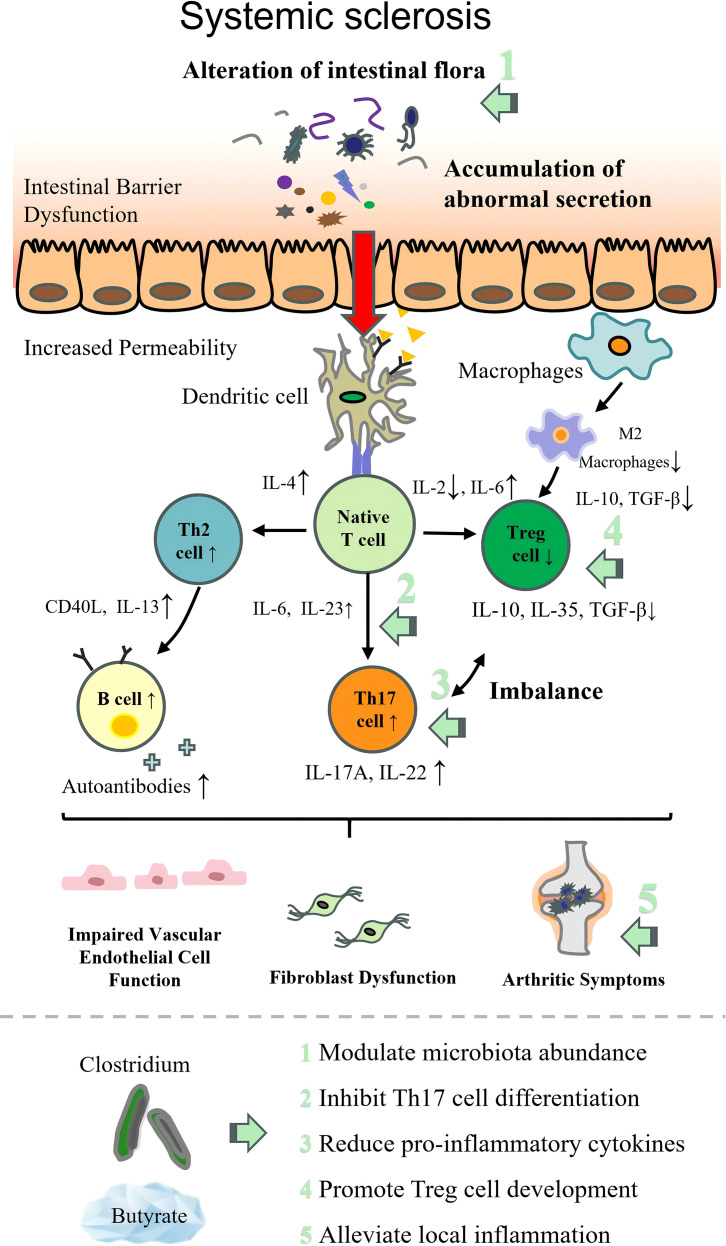
Pathogenesis of systemic sclerosis with therapeutic targets of beneficial *Clostridium*. LPS, lipopolysaccharide; SCFAs, short-chain fatty acids; Th2 cell, T helper 2 cell; Th17, T helper 17 cell; Treg, regulatory T cell; IL, interleukin; CD40L, CD40 ligand; TGF-β, transforming growth factor β.

## 4 Conclusions and perspective

Inadvertently, the field of research is gradually focusing on smaller organisms, microbiomes are making their way onto the research “scene,” and the intestinal microbiota, mainly nonculturable bacteria, play a role in improving a healthier intestinal ecology by exerting their innate local and adaptive immune superiority. Exquisitely designed studies on the role of *Clostridium* and their metabolism in ADs have made it a promising strategy for immunotherapy.

This review systematically synthesizes the pathogenic role of gut microbiota dysbiosis in eight major ADs and highlights the central protective role of beneficial *Clostridium* species. Consistent evidence across clinical, preclinical, and *in vitro* studies confirms that key *Clostridium* taxa, predominantly *CB*, *FP*, *Clostridium* leptum, and *Clostridium* clusters IV/XIVa, are uniformly depleted in these ADs. This depletion is tightly linked to the breakdown of immune homeostasis, intestinal barrier dysfunction, and uncontrolled systemic inflammation, underscoring *Clostridium*’s potential as a core target for microbiota-based immunotherapy.

The therapeutic effects of *Clostridium* and their metabolites (e.g., SCFAs, butyrate, PCS, and vitamin K2) are mediated through a synergistic network of mechanisms, with precise correction of immune dysregulation standing as the most pivotal value. In innate immunity, *Clostridium* modulates macrophage polarization (shifting from pro-inflammatory M1 to anti-inflammatory M2), induces tolerogenic dendritic cell phenotypes to suppress excessive antigen presentation, regulates inflammatory body activity and neutrophil hyperactivation, and modulates ILC subsets to reinforce mucosal defense. For adaptive immunity, it restores the balance of T-cell subsets, promoting regulatory Treg differentiation, inhibiting pathogenic Th17 and autoreactive CD8^+^T cell activation, and regulating Tfh cell function. Complementing immune regulation, *Clostridium* rebalances gut microbiota homeostasis by enriching beneficial taxa and inhibiting pro-inflammatory bacterial overgrowth and reinforces intestinal barrier integrity by upregulating tight-junction proteins and mucin secretion, thereby blocking the translocation of pathogens and antigens that initiate systemic autoimmunity.

These conserved mechanisms are accompanied by disease-specific adaptations that address the unique pathological features of each AD: alleviating intestinal inflammation and reducing recurrence in IBD; mitigating joint bone erosion and autoantibody production in RA; protecting renal function and suppressing multi-organ inflammation in SLE; preserving pancreatic β-cell viability in T1D; relieving exocrine gland inflammation in pSS; attenuating vascular lesions in vasculitis; regulating hepatic immune responses and bile acid metabolism in PBC; and inhibiting skin and visceral fibrosis in SSc.

Building on these core therapeutic mechanisms, *Clostridium*-based interventions exhibit clear stratified clinical positioning in ADs, with their therapeutic significance defined by evidence maturity and disease-specific pathological characteristics, serving as a complement rather than a substitute for conventional therapies. For IBD, *Clostridium*-based strategies can act as adjuvant therapy to prevent recurrence in remission phases and as supplementary intervention in the acute phase. For RA and T1D, given limited evidence from small-sample clinical studies or preclinical models, these interventions are currently in the exploratory stage and not recommended to replace conventional immunomodulators. However, they can be used as exploratory adjuncts in clinical trials to mitigate inflammation and slow disease progression. For other ADs, therapeutic potential has mainly been validated in animal or *in vitro* models, whereas human clinical data remain scarce and limited to small cohorts; thus, clinical application awaits further verification of safety and efficacy, and they are not recommended for routine clinical use at present but are considered as exploratory research directions. Across all disease phases of various ADs, *Clostridium*-based interventions can serve as adjuvants: In the acute phase, they assist conventional therapies in alleviating inflammation by reinforcing the intestinal barrier and suppressing pro-inflammatory pathways; in the remission phase, they maintain gut microbial balance and promote immune tolerance to reduce recurrence risk. Their core value lies in synergizing with conventional treatments to enhance efficacy and reduce adverse effects, addressing unmet needs in ADs such as long-term remission maintenance and slowing early disease progression, without replacing core first-line therapies.

Nevertheless, potential risks of *Clostridium*-based interventions should not be overlooked in clinical practice. For patients requiring long-term *Clostridium* intervention during remission, attention should be paid to the cumulative risk of native gut microbiota disruption. Individual differences in gut microbiota baseline result in heterogeneous therapeutic responses, with some patients possibly showing no improvement or even adverse reactions. Notably, when combined with immunosuppressive drugs, *Clostridium*’s immunomodulatory effects may enhance immune activity and compromise drug efficacy, requiring close monitoring of immune function and timely dosage adjustments. Additionally, the safety data of *Clostridium* interventions in special populations such as children, pregnant women, and patients with severe immune deficiency are still lacking, and routine use is not recommended temporarily. These risks highlight the need for individualized assessments before intervention, standardized dosing and duration, and long-term safety monitoring to ensure clinical applicability and minimize potential harms.

Despite these promising findings and clear clinical positioning, current studies on beneficial *Clostridium* in ADs still exhibit significant common limitations that restrict clinical translation. First and foremost, there is a distinct imbalance in the level of evidence. Beyond IBD and RA, most research on diseases such as pSS, SSc, and PBC has relied heavily on rodent models. However, inherent differences exist in gut microbiota composition and immune cell response patterns between rodents and humans, leading to insufficient cross-species validation of core mechanisms. Meanwhile, human clinical data are generally characterized by small sample sizes and short follow-up periods, with inadequate adjustment for confounding factors such as diet and concurrent medications, making it difficult to confirm the long-term efficacy and safety of *Clostridium* interventions. Secondly, numerous flaws are present in study designs. Cross-sectional studies account for a relatively high proportion, which hinders the verification of causal relationships between changes in *Clostridium* abundance and disease onset. Some studies employ mixed-strain probiotic formulations, precluding the isolation of the independent therapeutic effects of *Clostridium*. Furthermore, heterogeneity in detection methods leads to poor comparability of results. Differences in resolution between 16S rRNA sequencing and metabolomics result in poor comparability in quantifying *Clostridium* abundance. Additionally, standardized clinical detection protocols for core indicators are lacking, which exacerbates discrepancies in study outcomes. Notably, the insufficient depth of mechanism validation is equally prominent. Most studies merely confirm the association between *Clostridium*/its metabolites and classical signaling pathways, without identifying the direct targets of action on target cells. Some mechanistic research relies on non-disease-specific models or tumor cell lines, and the applicability of their conclusions in clinical settings of autoimmune diseases remains to be verified. These overlapping limitations highlight an urgent need for further validation through large-scale, single-strain, long-term follow-up multicenter RCTs.

These inherent study limitations also contribute to inconsistent research findings in the field. Most studies indicate an inverse correlation between the abundance of beneficial *Clostridium* and AD severity. However, a small number of studies have reported contradictory results. We consider that several factors may explain this discrepancy. First, strain-specific differences play a role. Different *Clostridium* subtypes have distinct metabolite profiles. Second, sample heterogeneity is a key factor. Dietary patterns and genetic backgrounds differ among populations from various regions. These differences influence gut microbiota baselines and responsiveness to interventions. Additionally, differences in detection methodologies and study designs should not be overlooked. This suggests that future studies should reduce heterogeneity-induced interference by clarifying strain characteristics, stratifying by disease stage, and standardizing detection protocols and experimental designs, thereby more accurately revealing the true association between *Clostridium* and ADs.

In summary, beneficial *Clostridium* and their metabolites exert multifaceted protective effects in eight major ADs by regulating gut-immune crosstalk, reinforcing intestinal barrier integrity, and alleviating disease-specific pathological damage, holding great promise as a complementary strategy for clinical immunotherapy. However, current research is constrained by uneven evidence quality, inconsistent findings due to multidimensional heterogeneity, and insufficiently clarified direct molecular targets. Future studies should prioritize large-scale, single-strain multicenter RCTs to validate long-term efficacy and safety, standardize detection protocols and experimental designs to reduce heterogeneity, and deepen mechanistic exploration of *Clostridium*/metabolite–target cell interactions. With advances in personalized microbiota profiling and precision intervention strategies, *Clostridium*-based therapies are expected to overcome existing translational gaps, providing new hope for improving the prognosis of patients with intractable autoimmune diseases.
