# Supplementary material for: Recent advances in the positive role of Clostridium in autoimmune diseases: as a crucial regulator of restoring immune-metabolic equilibrium
Source: Front Immunol. 2026 Mar 25;17:1730351. doi: 10.3389/fimmu.2026.1730351 (PMC13057458; doi:10.3389/fimmu.2026.1730351)
Supplement: Supplementary file 1 [file SupplementaryFile1.docx]

**Supplementary File 1**

**Literature Search Strategy**

We conducted a systematic literature search in four authoritative biomedical databases: PubMed, Embase, Cochrane Library, and Web of Science. The retrieval timeframe was set from January 2010 to July 2025. Additional eligible articles were supplemented by manually screening the reference lists of included studies and relevant high-impact reviews to ensure no important research was omitted.

The PubMed retrieval formula (combining MeSH terms and free words for comprehensive coverage) was as follows: (("*Clostridium*"[MeSH Terms] OR "*Clostridium*"[All Fields] OR "*Clostridium butyricum*"[All Fields] OR "*Faecalibacterium prausnitzii*"[All Fields] OR "*Clostridium cocleatum*"[All Fields] OR "*Clostridium nexile*"[All Fields] OR "*Clostridium saccharogumia*"[All Fields] OR "*Clostridium leptum*"[All Fields] OR "*Clostridium lavalense*"[All Fields] OR "*Clostridium cluster IV*"[All Fields] OR "*Clostridium cluster XIVa*"[All Fields] OR "*Clostridium cluster XIVb*"[All Fields] OR "*Clostridium cluster XVIII*"[All Fields] OR "*Clostridium cluster XI*"[All Fields] OR "Butyrate"[All Fields] OR "Short-Chain Fatty Acids"[All Fields]) AND ("Autoimmune Diseases"[MeSH Terms] OR "Autoimmune Diseases"[All Fields] OR "Inflammatory Bowel Disease"[All Fields] OR "ulcerative colitis"[All Fields] OR "Crohn’s disease"[All Fields] OR "Rheumatoid Arthritis"[All Fields] OR "Systemic Lupus Erythematosus"[All Fields] OR "Type 1 Diabetes"[All Fields] OR "Primary Sjögren's Syndrome"[All Fields] OR "Vasculitis"[All Fields] OR "Kawasaki Disease"[All Fields] OR "Henoch-Schönlein Purpura"[All Fields] OR "Primary Biliary Cholangitis"[All Fields] OR "Systemic Sclerosis"[All Fields])). Retrieval strategies for other databases adopted analogous logic, and adjusted to match each database’s specific indexing system.

1. Inclusion Criteria

The included studies should be confirmed with the following regulations. Study type: Original research, including clinical studies (cohort studies, case-control studies, randomized controlled trials), animal model studies (e.g., murine models of autoimmune diseases), and in vitro mechanistic studies. Research objects: Human autoimmune disease patients, autoimmune disease animal models (e.g., NOD mice for type 1 diabetes, DSS-induced colitis mice for inflammatory bowel disease), or immune-related cells (e.g., T cells, macrophages, dendritic cells, intestinal epithelial cells) [3]. Intervention/exposure: Beneficial Clostridium species (as single strains or mixed consortia), their metabolites (e.g., butyrate, propionate, p-cresol sulfate), or Clostridium-based probiotic formulations. Outcome measures: Primary outcome measures included ①changes in gut microbiota composition (e.g., abundance of beneficial Clostridium species); ②indicators of immune function (e.g., Treg/Th17 cell balance, cytokine levels); ③intestinal barrier integrity (e.g., tight junction protein expression, intestinal permeability).

1. Exclusion Criteria

The excluded studies were defined as follows: ①Non-original articles (e.g., reviews, meta-analyses, editorials, letters to the editor). ②Studies focusing on pathogenic Clostridium species (e.g., Clostridium difficile). ③Studies with unclear Clostridium strains, incomplete key data (e.g., lack of outcome indicators for immune function or disease severity), or unextractable core results. ④Duplicate publications (only the latest version with the most comprehensive data was retained).
